# Meteorin-like controls metabolic adaptations and physiological myocardial remodeling in pregnancy and lactation

**DOI:** 10.3389/fendo.2026.1823116

**Published:** 2026-06-24

**Authors:** Albert Blasco-Roset, Celia Ruperez, Artur Navarro-Gascon, Francisco Javier Godoy-Nieto, Fàtima Crispi, Francesca Crovetto, Eduard Gratacós, Francesc Villarroya, Tania Quesada-López, Anna Planavila

**Affiliations:** 1 Departament de Bioquímica i Biomedicine Molecular. Institut de Biomedicina de la Universitat de Barcelona (IBUB), Universitat de Barcelona, and CIBER Fisiopatología de la Obesidad y Nutrición (CIBEROBN), Barcelona, Catalonia, Spain; 2 BCNatal -Barcelona Center for Maternal-Fetal and Neonatal Medicine (Hospital Clinic and Hospital Sant Joan de Deu), Institut d’Investigacions Biomediques August Pi i Sunyer (IDIBAPS), University of Barcelona, Centro de Investigación Biomédica en Red de Enfermedades Raras (CIBER-ER), Barcelona, Catalonia, Spain; 3 Department of Infectious Diseases, Hospital de la Santa Creu i Sant Pau. Institut de Recerca de l’Hospital de la Santa Creu i Sant Pau. Carrer de Sant Quintí, Barcelona, Catalonia, Spain

**Keywords:** cardiac hypertrophy, cardiokine, gestation, lactation, lipid metabolism

## Abstract

**Introduction:**

The gestation–lactation period involves profound but reversible cardiac remodeling. Meteorin-like (Metrnl) has emerged as a regulator of cardiac metabolism in pathological settings, but its role in physiological reproductive adaptations remains unclear. This study aimed to define the contribution of Metrnl to cardiometabolic remodeling during the reproductive cycle.

**Methods:**

Circulating Metrnl levels were assessed in murine and human late-pregnancy models. Wild-type (wt) and Metrnl knockout (*Metrnl^-/-^*) mice were analyzed across gestation, lactation, and weaning to evaluate cardiac structure and metabolism.

**Results:**

Late pregnancy increased circulating METRNL, largely of placental origin. In wt mice, cardiac hypertrophy induced by pregnancy was further enhanced during lactation and fully reversed after weaning. These changes were associated with increased fatty acid metabolism during gestation and its suppression during lactation, paralleling cardiac Metrnl expression and normalizing after weaning. By contrast, *Metrnl^-/-^* mice showed exaggerated eccentric hypertrophy during gestation and lactation, with delayed regression post-weaning. This was accompanied by impaired metabolic flexibility and myocardial lipid accumulation, consistent with cardiac dysfunction.

**Conclusion:**

Metrnl is essential for physiological cardiometabolic adaptation during the reproductive cycle, coordinating metabolic remodeling to ensure appropriate and reversible cardiac changes.

## Introduction

Cardiovascular disease (CVD) is the primary cause of death in European women, with a higher deceased percentage than in men (1). However, there is still a gap in the general awareness of cardiovascular disease in the female population and particularly it is unknown to what extent physiological differences drive these disparities (2).

The physiologic experience of pregnancy is unique to females and requires a profound, but transient, adaptation of the cardiovascular system. During pregnancy the heart enlarges to compensate for the increased cardiac output and volume overload inducing an eccentric hypertrophy characterized by chamber enlargement and a proportional change in wall thickness (3, 4). This cardiac remodeling occurs with cardiac function being preserved (5) or slightly depressed during late pregnancy (6, 7), and, in contrast with pathological cardiac hypertrophy, lack of fibrosis and no induction of fetal gene expression (8–10). However, the underlying molecular mechanisms mediating this adaptive maternal cardiac remodeling are not well understood. Moreover, no study has addressed a comprehensive analysis of the impact of lactation and posterior weaning on the maternal heart plasticity, and only a few studies have explored lactation-independent post-partum consequences in heart (5, 11, 12). A proper understanding of the molecular mechanisms responsible for cardiac physiological adaptations are important because failure of the heart to properly adapt during the gestation/lactation period could predispose the mother to short- and long-term adverse cardiac consequences and may also have an impact in offspring development.

During pregnancy, the hypertrophied heart exhibits increased fatty acid oxidation (FAO) and decreased glucose utilization (11, 13–15) in contrast to pathological cardiac hypertrophy -usually accompanied by a switch from fatty acid to glucose metabolism. The systematic alterations observed in cardiac metabolism associated to the development of physiologic/pathologic cardiac hypertrophy suggest that they may play a causal role in the development of cardiac remodeling instead of being a consequence.

In this sense, we have recently shown that the secreted protein Meteorin-like (Metrnl) promotes cardiac fatty acid metabolism thus inhibiting pathologic hypertrophy development (16). Metrnl is a cytokine expressed and secreted by different organs, including the heart where it is produced by cardiomyocytes but also by immune cells (17, 18). Metrnl also protects against cardiac ischemia/reperfusion development (19). In humans, we have reported that METRNL circulating levels independently predict a worse outcome in myocardial infarction patients (20). Despite the relevance of Metrnl as an emerging actor in cardiac diseases, its role during the gestation/lactation period is completely unknown.

Here, we sought to determine the role of Metrnl during pregnancy, lactation, and weaning in relation to cardiac hypertrophy. We show that pregnancy increases systemic Metrnl levels -mainly due to placental and cardiac contribution- and induces eccentric cardiac hypertrophy, a phenomenon further increased during lactation and totally reversed upon weaning. However, Metrnl insufficiency (in *Metrnl^-/-^* mice) induced an exacerbated eccentric hypertrophy development and cardiac disfunction and a delayed post-weaning resolution. These findings indicate that Metrnl is a major mediator of the maternal cardiac adaptations occurring during pregnancy and lactation.

## Materials and methods

### Human samples

Maternal third trimester plasmatic samples and snap-frozen placental tissue were retrieved from the non-intervention group of the IMPACT BCN Randomized Clinical Trial (NCT03166332), which involved singleton pregnancies including: non-complicated non-obese pregnant women with normal glycemic and insulin levels (n=43; age=30.9 ± 4.7) and obese pregnant women (BMI>30; n=58; age=31 ± 3.7). All samples (plasma and frozen placenta) derived from the same patient cohort with matched plasma and placental samples. Age-matched plasmatic samples from non-pregnant women (n=15, age=34 ± 4.6) were used as controls. Human tissues were obtained as previously described (21, 22).

### Animal study.

*Metrnl*-null mice (C57BL/6NTac-Metrnl^tm1a(KOMP)Wtsi^/WtsiH; EM:07966) were obtained from the Wellcome Trust Sanger Institute Mouse Genetics Project (Sanger MGP). Wild-type littermates were used as controls for all experiments with *Metrnl^-/-^*. All mice were housed at the same room with a 12h light/dark cycle from 8am to 8pm, a controlled 22°C room temperature and ∽50% humidity. Food and drinking water were disposed *ad libitum*. Wild-type and *Metrnl^-/-^* female mice were mated overnight with genotype-matched males. The control group (non-pregnant mice; NP) was adjusted to be age-matched at each time point. NP, pregnant mice at day 18 (E18), lactating mice at days 7 (L7), 14 (L14) and 21 (L21); and mice after weaning at days 7 (W7), 21 (W21), 35 (W35), and 63 (W63) were studied. Only mice with litters of 6 or more pups were included in the studies. Female mice were randomly assigned to each time-point of sacrifice (E, L or W) and this was only known by the responsible animal caretaker. To estimate sample size, a pilot study at lactating 14 (L14), the peak of lactation, was performed using wild-type and *Metrnl^-/-^* female mice. Mean and standard deviation (SD) of *Myh7*, *Acta1*, and *Nppa* gene expression (known canonical hypertrophy gene markers) were used to perform calculation of sample size by power analysis (23). A range of 5–7 mice per group was determined to be an optimum number of animals to attain statistical significance with 90% probability.

Mice were euthanized by bleeding via intracardiac puncture under 5% isoflurane anesthesia during the morning under no food or drink restriction; blood was collected in heparinized tubes and centrifuged (3000rpm, 7.5 min) for plasma preparation, whereas the heart was removed and weighed. Each heart was extracted and cut transversely at mid-height; one half was prepared for optic or electron microscopy, while the other was immediately snap-frozen in liquid nitrogen and stored at -80°C until processing.

### Longitudinal echocardiography in non-pregnant, pregnant, lactating, and weaned mice

At each time-point described above, mice were anesthetized with 4% isoflurane for induction and kept under 1.5% isoflurane during the procedure. Cardiac dimensions were assessed by echocardiography using a commercially available system (Vivid Q; GE Healthcare, Piscataway, NJ, USA) equipped with a 12-MHz linear probe. Ventricular dimensions were measured using M-mode scans across the left ventricle. The mean heart rates from the mice were always maintained over 450 bpm to ensure data reliability.

### Plasma metabolites and hormone levels

Glucose and triglyceride levels were measured in fresh blood samples using an Accutrend Technology system (Roche Diagnostics, Basel, Switzerland). From the prepared plasma after sacrifice, Metrnl/METRNL plasma levels were measured using a mouse/human Metrnl/METRNL ELISA kits (R&D systems). Insulin was quantified using a Multiplex system (MADKMAG-HK, Millipore). Finally, from snap-frozen cardiac samples, lactate intracardiac levels were measured using #MAK329 kit (Sigma-Aldrich) following the manufacturer’s instructions.

### Heart histology

Each heart was extracted and cut transversely at mid-height. One half was fixed in 4% formaldehyde, embedded in paraffin, and cardiac slices were stained with hematoxylin-eosin (H&E) for morphological analysis (cardiomyocyte size, using the ImageJ software) and with Masson’s trichrome staining for the detection of fibrosis, and then examined by light microscopy. Else, the mid-height hearts were embedded in OCT compound (Tissue-Tek), frozen on dry ice and stored at -80 °C for oil red O (ORO) staining for lipid screening. Tissue sections from the samples included in this study were processed by the HCB-IDIBAPS Biobank (B.0000575), integrated in the Platform ISCIII Biobanks and Biomodels and the Histopathology Facility at the Institute for Research in Biomedicine (IRB).

### Image quantification for cardiomyocyte cross-sectional area

Micrographs from axial histological sections of hearts stained with H&E were captured using a Motic AE2000 microscope with a Moticam 5.0 MP camera (Motic Instruments INC, Richmond, BC, Canada), as.tiff files, and were imported into the ImageJ software [Wayne Rasband at the National Institutes of Health (NIH)] and a manually curated estimation of the fibers size was performed. The measurements obtained were subsequently used for comparison.

### Electron microscopy

Left ventricle samples sized 8mm^3^ or less were fixed in 2% paraformaldehyde and 2.5% glutaraldehyde diluted in 0.1M phosphate buffer (pH=7.4). Samples were processed by CCiTUB (Universitat de Barcelona), and were postfixed in 1% osmium tetroxide (OsO4) and 0.8% potassium ferricyanide (K3Fe(CN)6). Further, they were dehydrated with serial acetone dilutions and finally embedded in Spurr resin. 70nm-thick sections were sliced with Leica UC7 ultramicrotome and contrasted with uranyl acetate and lead citrate. Finally, samples were visualized with a JEOL 1010 electron microscope and an Orius CCD camera. Quantitative analysis of lipid droplet number and area from electron microscopy images was performed using stereological methodology (24);

### Gene expression

Total RNA was isolated from snap-frozen left ventricular tissues using TriPure (Roche, Indianapolis, IN, USA). Reverse transcription of 500 ng of RNA was performed using a high-throughput cDNA synthesis kit. Lack of amplification in the absence of reverse transcriptase was systematically verified. qRT-PCR was performed using TaqMan probes (Applied Biosystems; **Supplementary Table 1**) in a 20 μL reaction mix which contained 1 μL of cDNA, 10 μL of TaqMan Universal PCR Master Mix (Thermo Fisher Scientific), and 250 nM of probes. The expression levels of each gene of interest were normalized to that of the reference gene PPIA or Ppia mRNA (human and mouse samples, respectively), which was evaluated and confirmed that Ct values were stable and non-variable across genotype and throughout the reproductive cycle (**Supplementary Figure 1**). The CT (2-ΔCt) comparative method was used for normalization.

### Single-cell RNA sequencing data analysis of placenta

Publicly available single-cell RNA sequencing (scRNA−seq) datasets for mouse and human placenta (GEO accessions GSE156125 and GSE270170) were accessed to obtain transcriptomic insights (25, 26). Data preprocessing followed a modified version of an open-access R pipeline hosted on GitHub (IRCGP-Lab/Macrophage-heterogeneity-after-MI) (27).

Data were processed with Seurat 3.2.0 in R. Low-quality cells (<500 genes or >10% mitochondrial reads in mice; <200 genes or >20% in humans) were excluded before normalization and variable gene selection via variance stabilizing transformation. Datasets were integrated using canonical correlation analysis (“FindIntegrationAnchors” and “IntegrateData”). Principal component analysis and shared nearest neighbor clustering (top 50 PCs for mice, 30 for humans) resulted in 49 mouse and 29 human cell clusters, as visualized using UMAP. Cell types were identified with SingleR (28) (version 1.2.4) and known markers, yielding 12 mouse and 6 human cell categories. Metrnl expression was normalized, mapped to clusters, and visualized with FeaturePlot using Z−scores and fold−change analysis of Metrnl-positive trophoblast, endoderm, decidual, and immune cells.

### Protein expression and enzymatic assays

Total protein from snap-frozen cardiac tissue was extracted using the MILLIPLEX MAP lysis buffer supplemented with PMSF and protease inhibitors. Western blotting was performed on 25 µg total protein per sample, resolved on 12% SDS–PAGE gels and transferred to PVDF membranes (Millipore). Ponceau staining confirmed equal protein loading. Target proteins included Pdk4 (ab214938, Abcam) and Mct4 (ab74109, Abcam), detected at 1:1000 dilution. In parallel, pyruvate dehydrogenase (PDH) enzymatic activity—a key marker of oxidative metabolism—was determined using the Abcam microplate-based enzyme activity assay (ab109902). Together, these analyses linked molecular signaling with metabolic adaptation in cardiac tissue.

### Statistical analysis

The results are presented as the mean ± SEM. The Student’s t-test, Mann-Whitney test with Dunnett’s *post-hoc* correction, or two-way ANOVA with Sidak’s *post-hoc* test were applied. Analyses were performed using the GraphPad Prism software. A p-value of less than 0.05 was statistically significant. For scRNA-sequencing R console, through RStudio, was used.

### Study approval

Human studies were approved by the Ethics Committee of Hospital Clinic (HCB/2016/0830) and was conducted in accordance with the Declaration of Helsinki. All participants gave signed consent after being fully informed of the goals and characteristics of the study. Details of the study population and design are published elsewhere (29). Animal experiments were performed in accordance with European Community Council directive 86/609/EEC and 2010/63/EU guidelines of the European Parliament on the protection of animals used for scientific purposes and were approved by the Animal Experimentation Ethics Committee (CEEA) of the University of Barcelona (PJ292/22).

### Data availability

Data used for mice and human scRNA-sequencing analysis are publicly available in Gene Expression Omnibus (GEO); accession number GSE156125 and GSE270170, respectively.

## Results

### Systemic maternal METRNL/Metrnl levels are increased during late pregnancy in humans and mice

**Figure 1:**
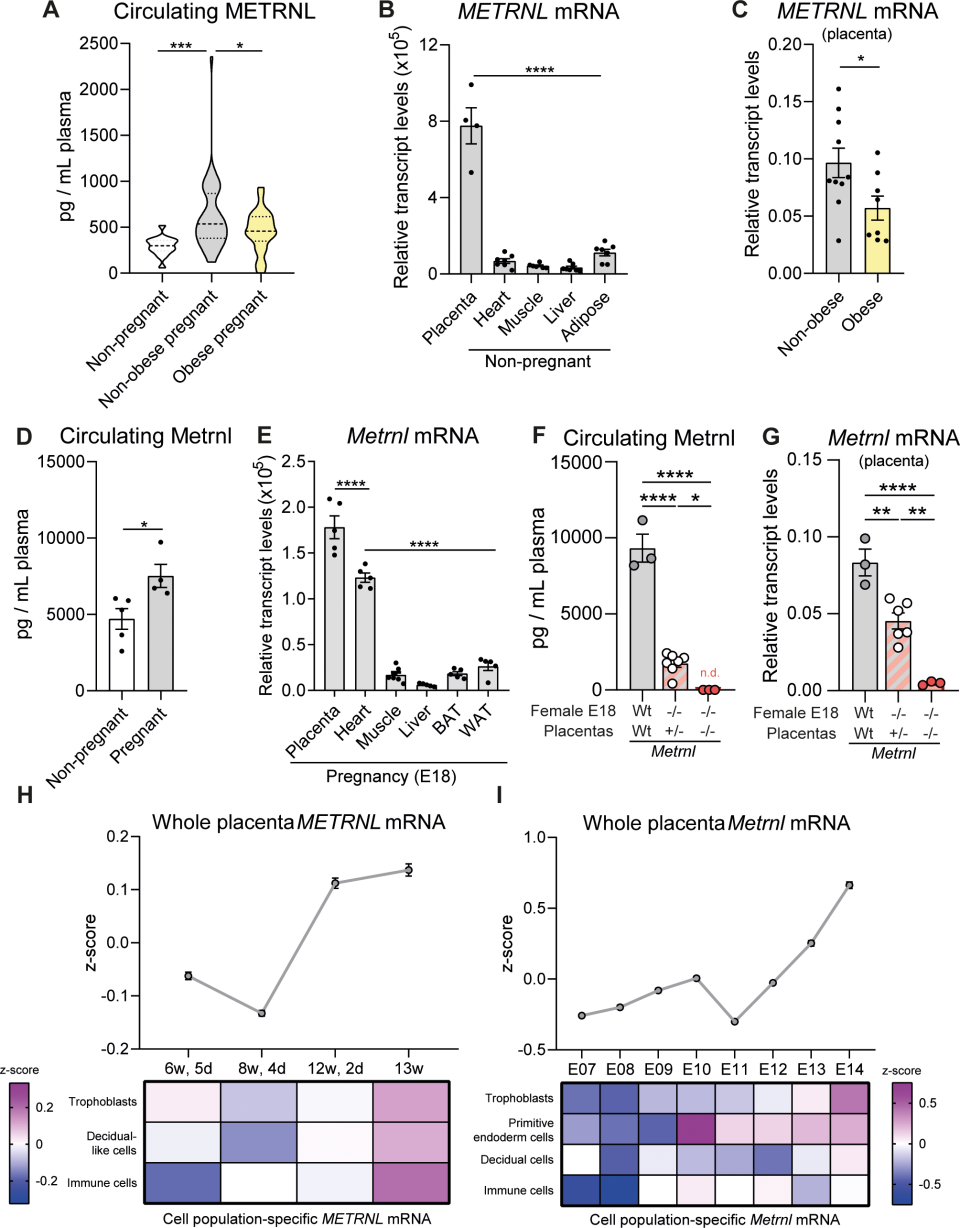
METRNL regulation in human and mice late pregnancy. Age-matched women were recruited, and **(A)** plasma levels of METRNL were estimated in the following human cohorts: non-pregnant (n=15), pregnant (n=43), and obese pregnant (n=58) women. ***p<0.001 compared to non-pregnant. **(B)** mRNA expression levels of *METRNL* were estimated in human samples from heart (n=7), liver (n=8), adipose tissue (n=7), and muscle (n=6) coming from non-pregnant individuals and placenta (n=4). **(C)** mRNA expression levels of *METRNL* in human placentas from non-obese pregnant (n=10) and obese pregnant (n=8) women were quantified. Levels of expression in human tissues were normalized to those of the reference gene PPIA. Results are presented as the mean ± SEM; one-way ANOVA and Sidak’s *post-hoc* corrections were applied to figures A and B; t-Student test was applied to figure **(C)** ^*^p<0.05 compared to non-pregnant and non-obese pregnant. ^****^p<0.0001 compared to placenta. Wild-type female mice were mated overnight, and age-matched non-pregnant mice served as controls. **(D)** Plasma levels of Metrnl in non-pregnant and pregnant female mice (n=5 mice/group) were quantified. **(E)** mRNA expression levels of *Metrnl* in brown adipose tissue (BAT), muscle, liver, white adipose tissue (WAT), heart, and placenta of female mice at day 18 of gestation (E18; n=5 mice/group). Circulating Metrnl levels **(F)** and placental *Metrnl* mRNA expression **(G)** at E18 in pregnancies from wt × wt, *Metrnl^-^/^-^* × *Metrnl^-^/^-^*, and *Metrnl^-^/^-^* females crossed with wt males. Levels of expression in murine tissues were normalized to those of the reference gene *Ppia*. ** P< 0.01 compared to heterozygous, **** p< 0.0001 compared to WT. **(H, I)** Sn-RNAseq publicly available data of placentas from mice and human at different time-points were accessed and analyzed. **(H)** Whole mice placenta mRNA expression levels of *Metrnl* at the different time-points of pregnancy and the expression profile of the main contribution cell types to this expression are shown. **(I)** Whole human placenta mRNA expression levels of *Metrnl* at the different time-points of pregnancy and the expression profile of the main contribution cell types to this expression are shown. Results are presented as the mean ± SEM; t-Student test was applied to figure D and one-way ANOVA and Sidak’s *post-hoc* corrections were applied to figure **(E)** ^*^p<0.05, compared to non-pregnant; and ^****^p<0.0001 compared to heart.

To determine the role of METRNL during pregnancy, we analyzed the maternal circulating levels of METRNL on a subset of samples from human pregnancies at the third trimester (subdivided into non-obese and obese) compared to non-pregnant women (**Figure 1A**; **Supplementary Table 1**). Maternal circulating levels of METRNL were significantly increased in non-obese non-complicated pregnancies as compared to non-pregnant women. However, METRNL levels were significantly reduced in the obese pregnant similar to the concentrations of non-pregnant women. We further analyzed *METRNL* mRNA expression levels in human heart, liver, white adipose tissue (WAT), and skeletal muscle obtained from lean, non-gestational individuals, given the limited availability of tissue samples from pregnant women, apart from the placenta. This analysis revealed that the human placenta exhibits the highest levels of *METRNL* expression (**Figure 1B**). In addition, when we compared the placentas from non-obese and obese pregnancies, we found that the gene expression levels of *METRNL* were significantly reduced in placental tissue from obese compared to non-obese pregnancies (**Figure 1C**). Collectively these data point out to the placenta as the major contributor to the systemic METRNL levels during pregnancy.

Next, we analyzed the plasma levels of Metrnl in non-pregnant and late pregnant mice (day 18, E18), and we found increased levels of Metrnl during pregnancy (**Figure 1D**). Moreover, analysis of *Metrnl* expression across multiple murine tissues during pregnancy revealed that the placenta exhibited the highest levels of *Metrnl* expression, followed by the heart, which also showed increased *Metrnl* expression during pregnancy (**Figure 1E**). Notably, in contrast to the human dataset, where tissues were obtained from non-pregnant individuals, the murine samples were collected from pregnant mice at E18.

To further assess the contribution of the placenta to circulating Metrnl levels during pregnancy, we crossed Metrnl knockout (*Metrnl^-^/^-^*) female mice with wild-type (wt) male mice to generate pregnancies with a heterozygous placenta in a *Metrnl^-^/^-^* maternal background. We then measured circulating Metrnl levels and placental *Metrnl* expression at E18 (**Figures 1F, G**). Metrnl was detectable in both the circulation and placenta of *Metrnl^-^/^-^* females at E18, although at lower levels compared to wt mice. These findings demonstrate a non-negligible contribution of the placenta to circulating Metrnl levels during pregnancy. Relevantly, Metrnl circulating levels were product of a hemizygous dose of placental Metrnl. Therefore, a full diploid dose would be expected to provide even higher levels of circulating, maternal Metrnl.

To evaluate the temporal and cellular contribution of the placenta to METRNL levels, we accessed publicly available datasets of mice (GSE156125, **Figure 1H**) and human placenta (GSE270170, **Figure 1I**). The analysis of these data showed a time-dependent induction in the expression of *Metrnl* mRNA levels in the whole placenta in both human and mice. Late pregnancy placentas showed the highest levels of expression of *Metrnl* and, when exploring the cell contribution of this induced mRNA expression, we discovered that the main cell types contributing to these inductions were the trophoblasts, decidual cells, primitive endoderm cells, and immune cells. Furthermore, UMAP of cell populations of the murine placenta revealed that the cell types showing the highest levels of *Metrnl* expression and greater presence in the tissue were the trophoblasts and decidual cells (cell type representation in the dataset: 18.27% for Trophoblasts, 20.95% for Decidual cells, 22.38% for immune cells and 2.01% for Primitive endoderm cells **Supplementary Figure 2**).

These results collectively indicate that the plasma protein levels of Metrnl are increased during pregnancy -mainly due to the placental contribution- and are reduced in females living with obesity.

### Metrnl^-/-^ female mice develop a higher degree of cardiac hypertrophy and fibrosis during gestation and lactation

**Figure 2:**
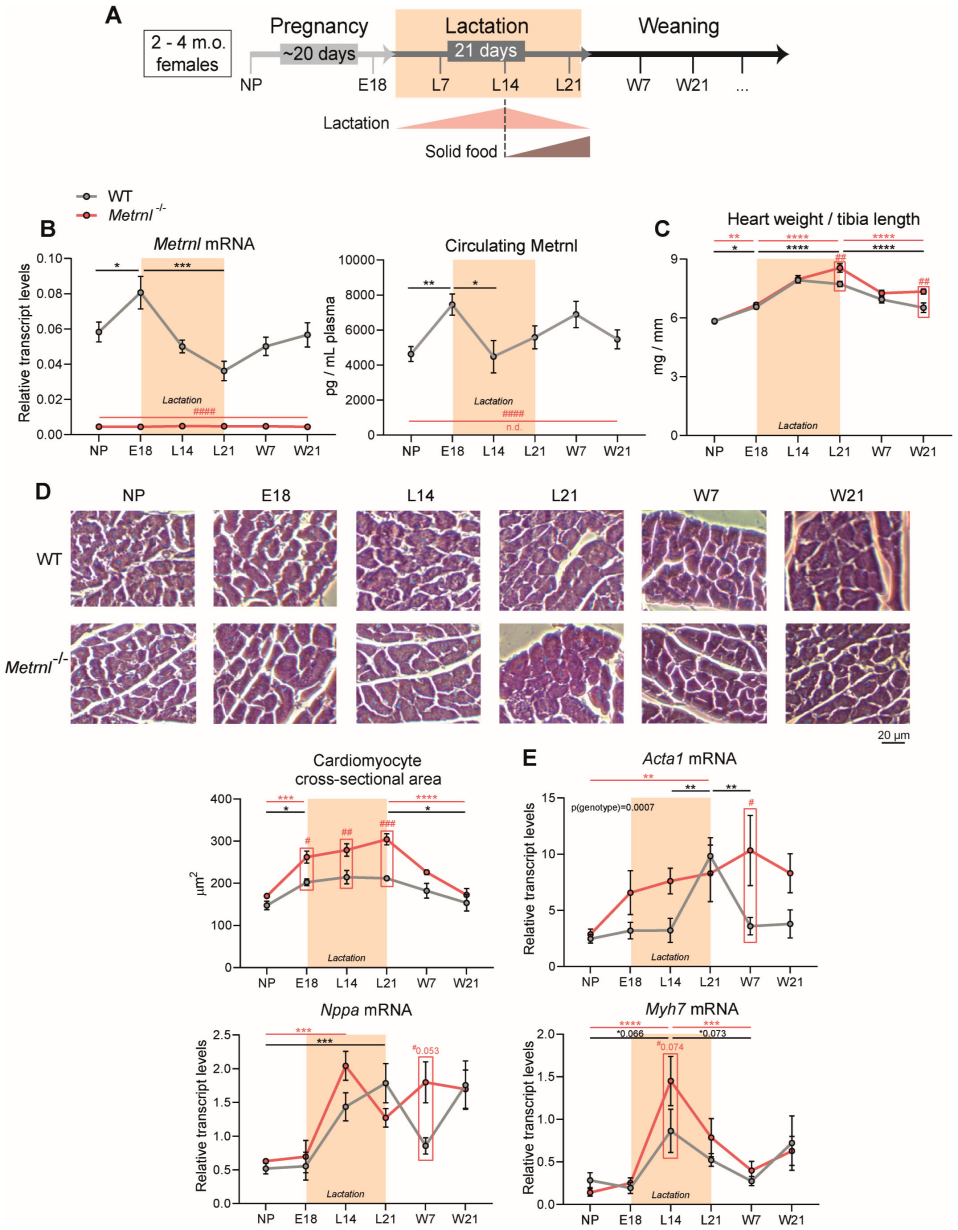
*Metrnl*-null mice develop enhanced cardiac alterations in pregnancy and lactation. Wild-type and *Metrnl^-/-^* female mice were mated overnight. Age-matched non-pregnant (NP) female mice served as controls. NP, pregnant mice at day 18 (E18), lactating mice at days 7 (L7), 14 (L14) and 21 (L21); and mice after weaning at days 7 (W7), 21 (W21), 35 (W35), and 63 (W63) were studied. **(A)** Schematic representation of the murine experimental model of pregnancy, lactation, and recovery in wild-type and *Metrnl^-/-^* female mice (n=5-13) is illustrated. In wild-type mice, **(B)** *Metrnl* mRNA expression in the left ventricle of the heart (LV) and plasma levels of Metrnl in NP, E18, L14, L21, W7, and W21 mice were estimated. Results are presented as the mean ± SEM; One-way ANOVA and Sidak’s *post-hoc* corrections were applied, ^*^p<0.05, ^**^p<0.01, and ^***^p<0.001 compared to E18; sample size = 5-7. In wild-type and *Metrnl^-/-^* mice, **(C)** differences in ratio of heart weight (mg) to tibia length (mm) (HW/TL) in NP, E18, L14, L21, W7, and W21 mice were assessed. **(D)** Images of histological sections used to quantify cardiomyocyte (CM) cross-sectional area (CSA) in the left ventricular wall from NP, E18, L14, L21, W7, and W21 mice, (n=3); scale bar 20µm. **(E)** LV heart mRNA expression of hypertrophy gene markers: *Acta1*, *Nppa* and *Myh7*. Results are presented as mean ± SEM. Data were analyzed by two-way ANOVA followed by Sidak’s *post-hoc* test. In black, comparisons for wt mice and in red comparisons for *Metrnl^-/-^* mice; ^*^p<0.05, ^**^p<0.01, ^***^p<0.001, and ^****^p<0.0001 compared to indicated time-points, same genotype. ^#^p<0.05, ^##^p<0.01, and ^###^p<0.001 for genotype comparisons at a given time-point; genotype effect is listed at the upper left corner of the graph when statistically significant; sample size = 5-7.

Considering the regulatory display of METRNL/Metrnl observed during gestation in both humans and mice, and its downregulation in humans in conditions of obesity, we undertook the study of changes in Metrnl levels and expression during gestation, lactation and weaning in mice, and we also determined the alterations occurring in Metrnl knockout (*Metrnl^-/-^*) female mice at these conditions. For this purpose, we studied virgin (non-pregnant females, NP), pregnant mice at term (E18), lactating females at days 14 -peak of lactation- and 21 (L14 and L21), and mice 7 and 21 days after L21 weaning (W7 and W21) (**Figure 2A**). There were no differences between genotypes in pregnancy duration or litter size and pup’s body weight at delivery or weaning (**Supplementary Table 2**).

First, we analyzed Metrnl regulation across the different periods (**Figure 2B**). We found that the cardiac expression levels of *Metrnl* were significantly increased during pregnancy (E18) compared to virgin female mice, significantly decayed during lactation (L14 and L21) compared to E18, and progressively recovered after weaning. Regarding the Metrnl circulating levels, they increased during pregnancy and declined during lactation (L14) returning to the levels of non-pregnant mice after weaning (**Figure 2B**). Concomitant to this regulation in cardiac Metrnl levels, *Kit* mRNA levels (the putative Metrnl receptor) were downregulated in pregnancy and lactation to start recovering progressively in parallel to the weaning of the pups (**Supplementary Figure 3A**). We confirmed the absence of both circulating Metrnl and cardiac Metrnl expression in *Metrnl^-/-^* mice. We further analyzed Metrnl gene expression in additional tissues that undergo substantial remodeling during the reproductive cycle, including brown and white adipose tissues (Supplemental **Supplementary Figure 3B**). In these tissues, we observed a distinct expression pattern compared to the heart, characterized by a decline during pregnancy followed by recovery in the postpartum period. Collectively, these results suggest that during pregnancy, the placenta and myocardium may represent the main sources of circulating Metrnl, while adipose tissues could contribute to circulating levels in the postpartum period.

As presented in **Table 1**, pregnant wt and *Metrnl^-/-^* mice exhibited similar increases in body weight relative to non-pregnant mice. During the lactation/weaning period the only difference observed was at L21 when *Metrnl^-/-^* mice presented a higher body weight compared to wt mice. Blood triglycerides (TGs) increased during pregnancy, decreased during L14, and recovered thereafter in both genotypes, although the TG levels were lower in basal *Metrnl^-/-^* mice. The circulating glucose behaves opposite to TG levels, decreasing during pregnancy and increasing post-delivery in both genotypes. Meanwhile, insulin levels, in fed conditions, were higher in late pregnancy -as previously described (30, 31)- and recovered after delivery in both genotypes.

**Table 1:** Body weight and plasmatic metabolite profile in pregnant mice.

	Non-pregnant (NP)		Late pregnancy (E18)		Peak of lactation (L14)	
	Wt	*Metrnl^-/-^*	Wt	*Metrnl^-/-^*	Wt	*Metrnl^-/-^*
Body weight (g)	24.24 ± 0.80	22.44 ± 0.69	38.41 ± 1.31 ^****^	38.17 ± 1.35 ^****^	31.09 ± 0.47 ^****,,####^	31.79 ± 1.06 ^****,,####^
TG (mg/dL)	191.5 ± 10.5	117.3 ± 6.6 **^++++^**	210.4 ± 15.1	162.6 ± 12.7	105.5 ± 7.1^****,,####^	113.8 ± 5.3 ^#^
Glucose (mg/dL)	167.8 ± 5.1	168.3 ± 3.5	127.5 ± 6.3 ^***^	137.8 ± 10.1 ^*^	159.4 ± 5.7^##^	146.3 ± 8.8
Insulin (pg/mL)	816.4 ± 218.6	606.9 ± 117.8	1490 ± 328.7	1405 ± 314.4 ^*^	918.2 ± 153.5	498.8 ± 53.2 ^##^
	Lactation day 21 (L21)		Weaning day 7 (W7)		Weaning day 21 (W21)	
	Wt	*Metrnl^-/-^*	Wt	*Metrnl^-/-^*	Wt	*Metrnl^-/-^*
Body weight (g)	27.11 ± 0.85 ^####^	31.76 ± 1.11 ^****,,###,^**^++^**	26.66 ± 0.56 ^####^	25.41 ± 0.59 ^####,&&&^	26.74 ± 0.53 ^####^	26.04 ± 0.96 ^*,####,&&&^
TG (mg/dL)	116.3 ± 7.6 ^****,,####^	154.5 ± 31.2	138.7 ± 6.1 ^**,,###^	118.0 ± 4.8	155.3 ± 13.0 #	152.2 ± 13.7
Glucose (mg/dL)	155.5 ± 6.8 ^#^	141.0 ± 3.5	154.7 ± 3.6 ^#^	185.3 ± 8.2 ^##,&&^	141.3 ± 10.3	184.5 ± 3.6 ^###,&&,^**^+++^**
Insulin (pg/mL)	744.8 ± 168.5	745.3 ± 192.8	464.7 ± 68.9	303.8 ± 24.6 ^##^	849.3 ± 170.5	828.7 ± 94.6 ^#^

Results are presented as mean ± SEM. Data were analyzed by 2-way-ANOVA (Sidak). * vs NP. # vs E18. & vs L21. ± vs WT. *** p< 0.001 vs NP same genotype , ****,pp< 0.0001 vs NP same genotype ##, ### pp<0.001 E18 same genotype, ####pp<0.0001 E18 same genotype, +++pp< 0.001 vs WT same timepoint, ++++pp< 0.0001 same timepoint, &&, &&& --> falta. Sample size: morphogenesis, n=5-8; circulating metabolites, n=5-15.

Next, we analyzed the hearts of these mice all over the gestation/lactation period (**Figure 2C**). We found that in wt mice the heart weight/tibia length ratio (HW/TL) was significantly increased during pregnancy, as expected. During the whole lactation period, the HW/TL enlargement was even higher compared with virgin female mice and even with late pregnant mice, a decrease just started after weaning and cardiac hypertrophy was completely reversed by weaning at day 21 (W21). Remarkably, in *Metrnl^-/-^* female mice the HW/TL ratio was further increased during the period of lactation relative to wild-type lactating mice and the post-weaning resolution was delayed with HW/TL ratios at day 21 (W21) significantly higher than virgin mice (**Figure 2C**). Consistent with data on heart enlargement, histological examination of H&E-stained left ventricular posterior wall (LVPW) and septum tissue sections revealed that the cardiomyocyte cross-sectional area (CSA) was larger in *Metrnl^-/-^* pregnant and lactating mice compared to wt mice (**Figure 2D**). Although both genotypes showed a significant increase in CSA in these conditions, the increase in *Metrnl^−/−^* was greater and the recovery to basal (virgin) levels is delayed (**Figure 2D**).

It has been shown that pregnancy-related cardiac hypertrophy does not induce markers of pathologic cardiac hypertrophy (8, 9) such as atrial natriuretic factor (*Nppa*), β-myosin heavy chain (*Myh7*), and α-actin (*Acta1*). Indeed, we confirmed that the mRNA expression levels of *Nppa, Myh7*, and *Acta1* were not significantly different in the hearts of pregnant and non-pregnant wt mice. However, we did find significant increases in the expression of these marker genes during the lactation period in mice: *Acta1* was significantly upregulated at L21 and *Nppa* was increased at L14 and L21 (**Figure 2E**). During the weaning period the expression levels of these markers in general returned to basal levels in wt mice. In *Metrnl^-/-^* female mice, *Nppa, Myh7*, and *Acta1* were over-expressed in heart relative to wt mice during the lactation period and in the case of *Acta1*, it did not return to basal levels at W21 day of weaning.

Fibrosis, which develops in the heart under pathologic conditions, is a hallmark of pathologic cardiac hypertrophy but is not seen during pregnancy-induced cardiac hypertrophy (8). Here, we quantified mRNA expression analysis of *Col1a1* (Collagen 1) and *Col3a1* (Collagen 3) and performed Masson’s trichrome staining to assess fibrosis in the hearts of pregnant wt and *Metrnl^-/-^* mice (**Supplementary Figure 3C**). We observed no differences in the mRNA expression levels of *Col1a1* and *Col3a1* during the pregnancy/lactation/weaning period in wt mice but in *Metrnl^-/-^* mice we observed a moderate, but significant, increase in these fibrotic markers all over the period. Although not abruptly enhanced, trichrome staining confirmed this moderate fibrosis in *Metrnl^-/-^* mice (**Supplementary Figure 3C**, micrographs). Based on these results, we conclude that during pregnancy/lactation period there is an induction of cardiac hypertrophy -not associated with changes in cardiac fibrosis- in wt mice, this cardiac hypertrophy during the reproduction cycle is greater in *Metrnl^-/-^* mice whereas signs of fibrosis enhancement occur in hearts from knock-out lactating mice.

Considering the anti-inflammatory and pro-angiogenic properties of Metrnl (16, 19, 32–34), we next analyzed the circulating protein levels of the pro-inflammatory type 1 cytokine Ccl2 and the cardiac expression levels of *Ccl2* and Tnfα (*Tnf*), the anti-inflammatory genes associated with type 2 cytokine signaling, *Arg1* and *Mrc1*, and the pro-angiogenic marker vascular endothelial growth factor α (*Vegfa*) (**Supplementary Figure 3D**). No relevant differences were observed for the expression of any of those genes due to gestation/lactation or to genotype indicating that they are not relevant actors in this type of physiological cardiac hypertrophy and in response to Metrnl deficiency.

### Lack of Metrnl impairs cardiac function during pregnancy/lactation and delays post-lactation resolution

**Figure 3:**
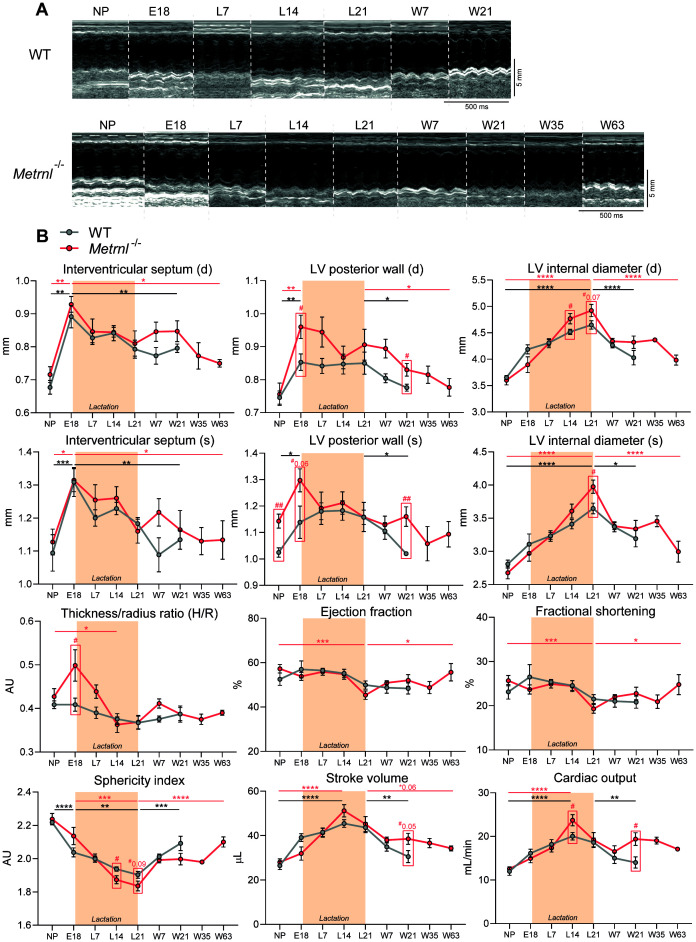
*Metrnl*-null female mice show a compromised cardiac function in pregnancy/lactation and display a delayed hypertrophy recovery. Wild-type and *Metrnl^-/-^* female mice were mated overnight. Mice were followed longitudinally since before mating and during pregnancy, lactation and weaning. **(A)** Representative M-mode echocardiography tracings from the left ventricle in NP, E18, L7, L14, L21, W7, W21, W35, and W63 of wild-type (upper panels) and *Metrnl^-/-^* mice (lower panels) are displayed (5millimeter/500millisecond). **(B)** Echocardiographic measurements from wild-type and *Metrnl^-/-^* female mice in **(A)** are shown. Results are presented as mean ± SEM. Data were analyzed by two-way ANOVA followed by Sidak’s *post-hoc* corrections. In black, comparisons for wild-type and in red comparisons for *Metrnl^-/-^* mice *p<0.05, **p<0.01, ***p<0.001, and ****p<0.0001 compared to indicated time-points, same genotype. #p<0.05 and ##p<0.01 for genotype comparisons at a given time-point; sample size = 5-7.

Next, to analyze cardiac function, we performed echocardiographic measurements during gestation, lactation and weaning (**Figure 3**; **Supplementary Table 3**). In wt animals we found increased levels of the internal ventricular septum (IVS) and the left ventricular posterior wall (LVPW) thickness in late pregnancy (E18) that were progressively reduced in the case of IVS or maintained for LVPW during the lactation period and completely returned to basal levels at W21. The end-diastolic and end-systolic left ventricular internal diameters (LVID) were higher in pregnant wt mice compared to their corresponding non-pregnant controls and continued increasing during the lactation period reaching the highest levels at L21 and decaying during the weaning period. We observed similar patterns for IVS, LVPW and LVID in *Metrnl^-/-^* mice but with higher values than wt mice and with a delayed post-lactation resolution. The sphericity index (SI) was significantly reduced in pregnant wt mice, continued dropping during lactation and was recovered after weaning indicating that there was some degree of eccentric hypertrophy/dilatation in wt mice during pregnancy that was exacerbated in the lactation period. A similar behavior but more pronounced was observed for *Metrnl^-/-^* mice. No difference was observed in the ejection fraction or fractional shortening in wt mice but a significant decrease of both was observed in *Metrnl^-/-^* mice at L21 indicating altered systolic cardiac function only in these mice. Finally, the stroke volume (SV) and the cardiac output (CO) significantly increased during pregnancy and lactation and showed a recovery to basal levels by W21 in both genotypes. Yet, these inductions in SV and CO were significantly higher in *Metrnl^-/-^* mice compared to wt mice (**Figure 3B**). In addition, the left ventricular mass (LVm) assessed by echocardiography was consistently higher in *Metrnl^-/-^* mice compared to wt controls from E18 throughout the reproductive cycle, with L21 representing the point of maximal divergence between groups (**Supplementary Table 3**). We conclude that in *Metrnl^-/-^* mice by L21, the increase in LVIDs without a change in LVIDd, combined with reduced EF and FS, indicates impaired systolic contraction without overt chamber dilation. This is consistent with early systolic dysfunction, likely associated with increased systolic wall stress and representing a transition toward decompensation.

Collectively these data indicate that whereas in wt mice pregnancy and lactation are associated to some degree of eccentric hypertrophy development and a complete resolution post-lactation, *Metrnl^-/-^* mice develop exacerbated eccentric hypertrophy during pregnancy and lactation, and a delayed hypertrophy reversion process.

### Mice lacking Metrnl have impaired cardiac fatty acid metabolism

**Figure 4:**
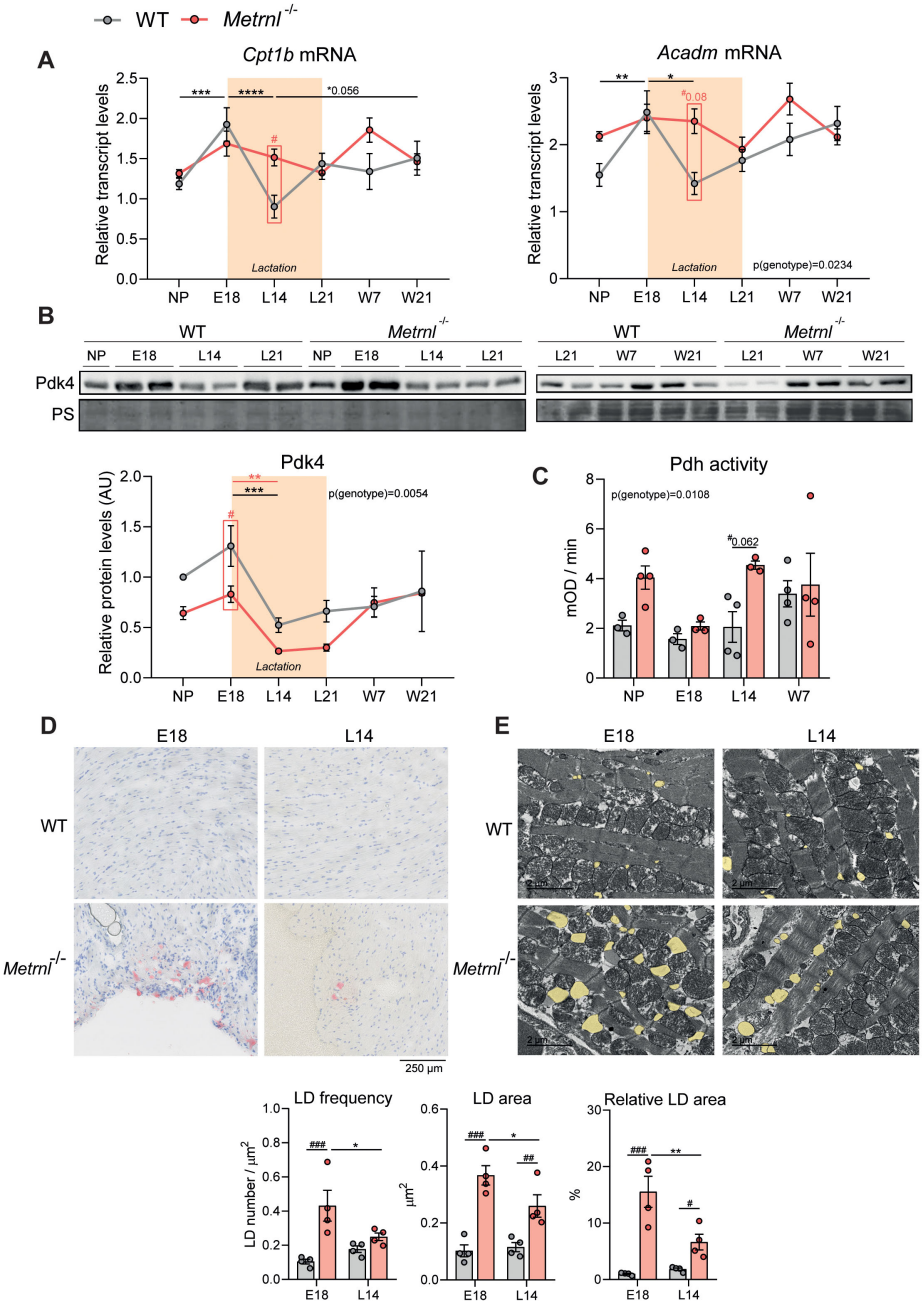
Lack of *Metrnl* in pregnancy/lactation results in impaired fatty acid metabolism. Wild-type and *Metrnl^-/-^* female mice were mated overnight; age-matched NP female mice served as controls. LV hearts form NP, E18, L7, L14, L21, W7, and W21 were processed. **(A)** mRNA expression levels of the lipid metabolism genes markers (*Cpt1b* and *Mcad*) were assessed, sample size = 5 - 7. **(B)** Representative images of western-blot membranes used to quantify Pdk4 protein levels in cardiac tissue; sample size = 3-4. **(C)** Protein extracts from cardiac samples of wild-type and *Metrnl^-/-^* female mice at NP, E18, L14 and W7 were used to determine the Pyruvate dehydrogenase (PDH) activity (mOD/min, sample size =4). **(D)** Representative histological sections of ORO-stained hearts from E18 and L14 mice wild-type (upper panel) and *Metrnl^-/-^* (lower panel) are shown (scale bar, 250µm, n=3). **(E)** Representative electron microscopy images and quantification of lipid droplets (LD) number and area. Results are presented as mean ± SEM. Data were analyzed by two-way ANOVA followed by Sidak’s *post hoc* test. In black, comparisons for wild-type and in red comparisons for *Metrnl^-/-^* mice: *p<0.05, **p<0.01, ***p<0.001, and ****p<0.0001 compared to indicated time-points, same genotype. #p<0.05, ## p<0.01 vs Wt L14; ### p<0.001 vs Wt E18 for genotype comparisons at a given time-point. Genotype effect is indicated [p(genotype)] at each graph when statistically significant.

Late pregnancy is characterized by increased fatty acid metabolism in the heart (8), but the cardiac metabolic profile during lactation and weaning is poorly characterized. Therefore, we next examined the mRNA expression levels of the genes involved in fatty acid metabolism carnitine palmitoyltransferase (*Cpt1b*) and medium-chain acyl-CoA dehydrogenase (*Acadm*, Mcad) in pregnant wt and *Metrnl^-/-^* mice. In wt mice (**Figure 4A**) pregnancy significantly increased the cardiac expression levels of all these genes which were significantly reduced thereafter the peak of lactation (day 14) and recovered after weaning. By contrast, *Metrnl^-/-^* mice did not significantly increased fatty acid oxidation genes due to gestation and the expression levels of *Mcad* and *Cpt1b* were significantly higher at L14 compared to wt mice.

Next, the protein levels of Pdk4, a known enzyme to contribute to the metabolic flexibility in substrate preference of cells, were examined (**Figure 4B**) and, in wt mice, we found an increase in Pdk4 levels during gestation that were significantly reduced during the lactation period and returned to basal levels upon weaning. In *Metrnl^-/-^* female mice, the profile of changes in Pdk4 during the gestation-lactation period was like that in wt mice, although levels were systematically lower.

Finally, we assessed the enzymatic activity of pyruvate dehydrogenase (PDH), the key enzyme determining fatty acid-versus-glucose oxidation, target of PDK4-mediated inhibition (**Figure 4C**). We found no significant differences in PDH activity due to gestation/lactation in wt mice, but PDH activity was significantly higher in *Metrnl^-/-^* mice compared to wt mice all over the reproductive cycle period suggesting metabolic shift in Metrnl-deficient hearts.

In addition to these observations, histological examination of the ORO-stained cardiac sections evidenced that pregnancy led to lipid accumulation in cardiomyocytes of *Metrnl^-/-^* pregnant mice but not in wt mice. This lipid accumulation was decreased, but not fully lost, during lactation in *Metrnl^-/-^* mice (**Figure 4D**). To validate this observation, we performed electron microscopy–based quantification of lipid droplets (LDs) (**Figure 4E**). Pregnant *Metrnl^-^/^-^* mice exhibited a significant increase in LD number and total LD area, an effect not observed in wt mice. Importantly, these changes persisted and were only partially resolved during lactation in *Metrnl^-^/^-^* mice.

Finally, we analyzed the expression levels of *Pparγ*, a key transcription factor in the regulation of lipid and glucose metabolism. While hearts from wt mice did not show differences in the expression of this transcription factor, the expression was induced with pregnancy in hearts of *Metrnl^-/-^* mice (**Supplementary Figure 4**). Moreover, when assessing the mRNA expression levels of canonical lipid metabolism marker genes (*Lpl*, *Pnpla2*, and *Dgat1*; **Supplementary Figure 4**), hearts from wt mice showed an induction in the expression of these markers with pregnancy, a repression with lactation, and a final re-establishment of basal mRNA expression levels after weaning. The regulation in these lipid metabolism markers across the reproductive cycle was not observed in *Metrnl*^-/-^ mice. Hearts from these knock-out mice fail to induce the expression of these lipid metabolism marker genes with pregnancy, on the contrary no regulation in the expression of these markers was observed during the reproduction cycle period (**Supplementary Figure 4**).

Collectively, these data indicate that while fatty acid oxidation in wild-type mice was tightly regulated -increasing during gestation and decreasing during lactation- *Metrnl^-^/^-^* mice failed to adapt to the metabolic demands of pregnancy and lactation, showing impaired fatty acid oxidation hence leading to lipid accumulation in the myocardium and compromising cardiac function.

### Metrnl^-/-^ mice present metabolic dysregulation during pregnancy and lactation

**Figure 5:**
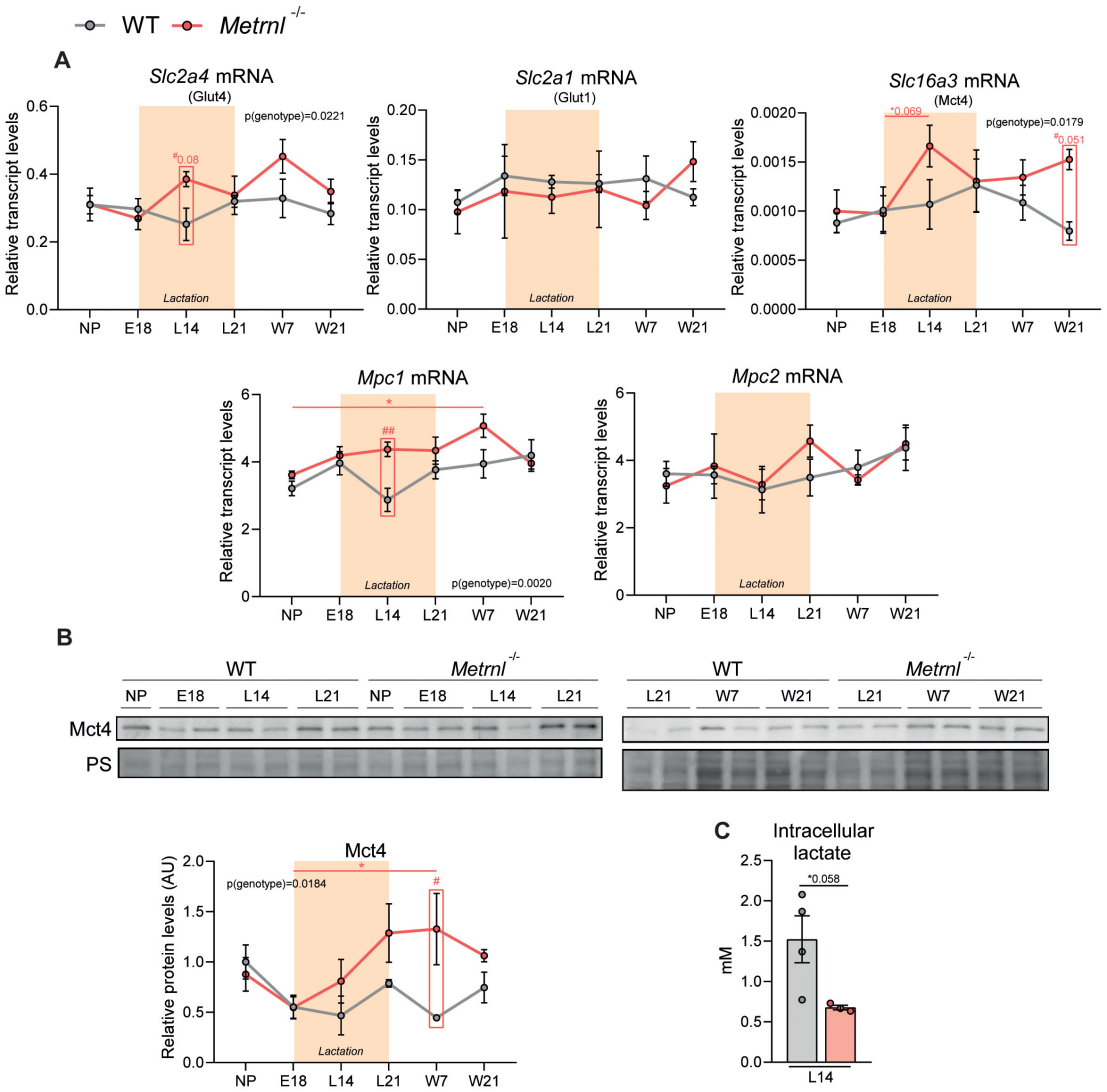
*Metrnl*-deficient female mice show a glucose-dependent metabolism in adjustment to pregnancy/lactation. Wild-type and *Metrnl^-/-^* female mice were mated overnight; age-matched NP female mice served as controls. LV hearts form NP, E18, L7, L14, L21, W7, and W21 were processed. **(A)** mRNA expression levels of the glucose metabolism genes markers (*Slc2a4*, *Slc2a1*, *Slc16a3*, *Mpc1*, *Mpc2*) were assessed (sample size = 5-7). **(B)** Representative images of western-blot membranes used to quantify Mpc4 protein levels in cardiac tissue are shown; sample size= 3-4. **(C)** Intracardiac lactate levels (mM) in L14 hearts from Wt and *Metrl*-/- mice are shown. Results are presented as mean ± SEM. Data were analyzed by two-way ANOVA followed by Sidak’s *post-hoc* test. In black, comparisons for wild-type and in red comparisons for *Metrnl^-/-^* mice; *p<0.05 compared to indicated time-points, same genotype. #p<0.05 and ##p<0.01 for genotype comparisons at a given time-point. Genotype effect is indicated (p(genotype)) at each graph when statistically significant.

In light of the inability of *Metrnl^-/-^* mice to modulate fatty acid metabolism we next analyzed the expression levels of genes involved in glucose transport and metabolism (**Figure 5A**): the glucose transporters *Slc2a1* (Glut1) and *Slc2a4* (Glut4), the mitochondrial pyruvate carriers *Mpc1* and *Mpc2*, and the monocarboxylate transporter-4 (*SIc16a3*, Mct4), which is believed to be the principal lactate exporter in cardiomyocytes and a hallmark of pathologic cardiac hypertrophy (35). No major changes in any of these genes in response to gestation/lactation in wt mice was observed. However, in *Metrnl^-/-^* mice we found that the expression levels of *Slc2a4* (Glut4)*, SIc16a3* (Mct4), and *Mpc1* were significantly higher compared to wt mice all over the gestation/lactation periods. Furthermore, assessment of Mct4 protein levels showed no differences in wt mice across the reproductive cycle but an induction in the expression of this lactate transporter in *Metrnl^-/-^* mice in lactation and further after weaning (**Figure 5B**). We next assessed lactate concentration during the peak of lactation (L14, **Figure 5C**) and we confirmed that intracellular lactate was decreased in the heart of the *Metrnl^-/-^* mice in agreement with the increased activity of PDH driving more pyruvate into the mitochondria in addition to the induced levels of Mct4 in these animals indicating an active removal of lactate from the cell. Overall, these findings suggest that *Metrnl^-/-^* mice have an altered cardiac metabolism during the gestation/lactation period.

### Pregnancy-induced cardiac hypertrophy is rapidly reversed after birth in the absence of breastfeeding

**Figure 6:**
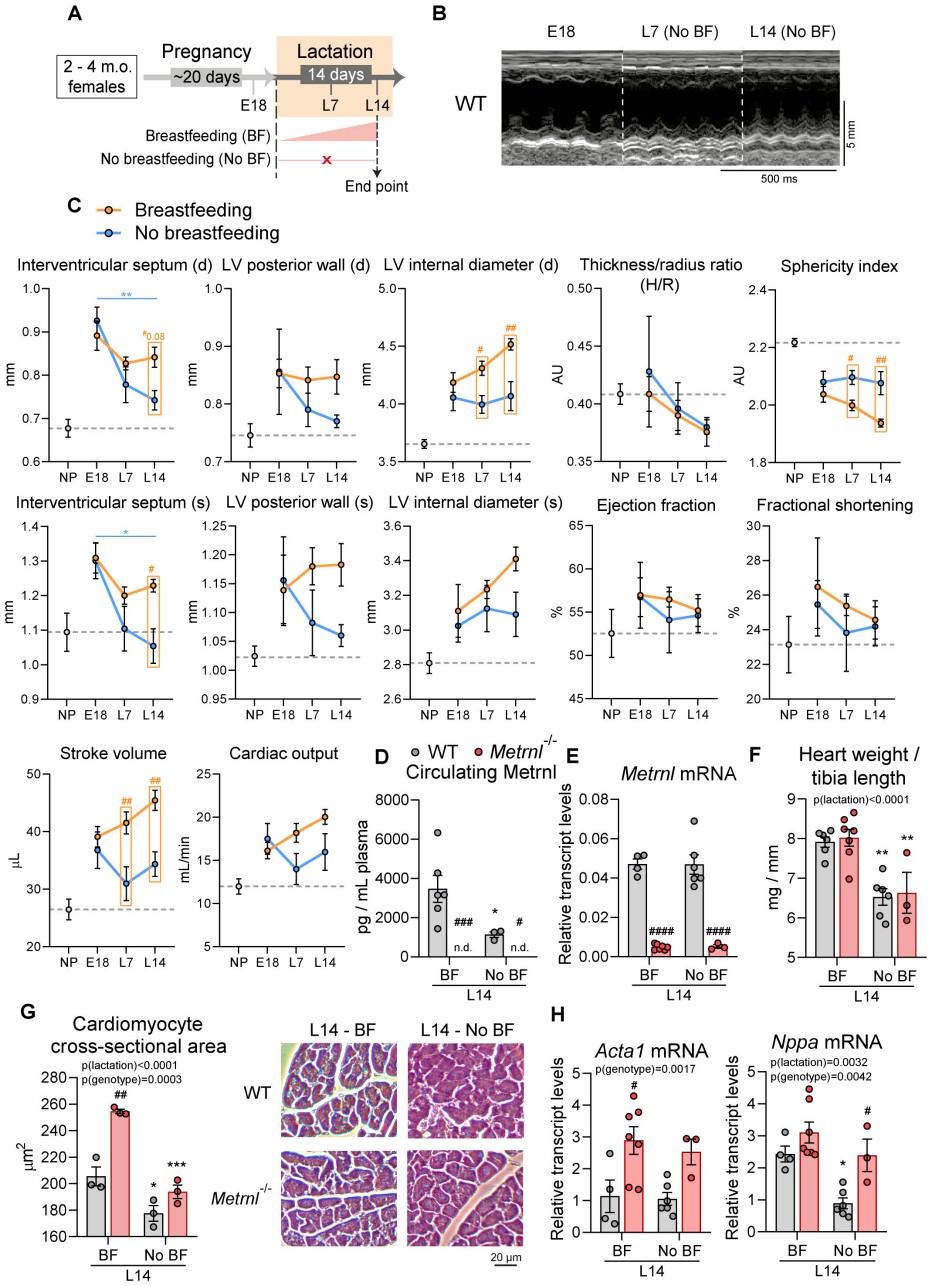
The absence of lactation reverses effects of pregnancy-modified cardiac dimensions. **(A)** Schematic representation of experimental design. Wild-type (wt) and *Metrnl^-/-^* female mice were mated overnight. Upon delivery, a subset of dams had pups removed (non-breastfeeding, non-BF), while the remaining dams breastfed normally (BF). Cardiac analyses were performed at embryonic day 18 (E18), lactation day 7 (L7), and lactation day 14 (L14). **(B)** Representative M-mode echocardiographic tracings of the left ventricle (LV) from non-BF wt mice at E18, L7, and L14 (bar = 5 mm/500 ms). **(C)** Quantification of echocardiographic parameters in non-BF wt mice corresponding to **(B)** (n = 4–7). **(D)** Circulating Metrnl levels in wt and *Metrnl^-^* mice at L14 under BF and non-BF conditions. **(E)** *Metrnl* mRNA expression in cardiac tissue from wt and *Metrnl^-^* mice at L14 under BF and non-BF conditions. **(F)** Heart weight to tibia length (HW/TL) ratio in wt and *Metrnl^-/-^* mice at L14. **(G)** Representative hematoxylin and eosin (H&E)-stained heart sections from wt (upper panel) and *Metrnl^-/-^* (lower panel) mice at L14 (20× magnification; scale bar = 20 µm). These images were used to quantify cardiomyocyte cross-sectional area (CSA) (n = 3). **(H)** Relative mRNA expression of hypertrophic markers *Acta1* and *Nppa* in LV tissue from wt and *Metrnl^-/-^* mice at L14. All data are presented as mean ± SEM. Statistical analysis was performed using two-way ANOVA followed by Sidak’s *post hoc* test. For **(C)**, *p < 0.05 and **p < 0.01 vs E18 within the same condition. For **(D–H)**, *p < 0.05, **p < 0.01, and ***p < 0.001 vs BF mice of the same genotype; #p < 0.05 and ##p < 0.01 indicate genotype differences under the same condition. Sample sizes are indicated in each panel (n = 3–7). Genotype effect is indicated [p(genotype)] at each graph when statistically significant.

To further assess the cardiac response associated to pregnancy and its reversion process, we next determined the behavior of the myocardium in no breastfeeding mice by removing the pups after birth (No BF) and analyzing the heart of female mice at 7- and 14-days post-partum (**Figure 6A**). For this assessment, we performed echocardiographic measurements during gestation and 7- and 14-days post-partum (**Figure 6B**; **Supplementary Table 4**), and we compared these mice with breastfeeding mice (BF) at the same 7- and 14-days post-partum (**Figure 6C**). We found that the IVS, LVPW, and LVID were significantly reduced in no BF compared to BF mice at L14, indicating that the cardiac geometry recovers faster after birth in absence of lactation. The cardiac function determined by EF and FS was also preserved in non-lactating dams. The SI was significantly increased in non-lactating female mice compared to lactating mice and the SV and CO were reduced (**Figure 6C**). All together these results indicate that pregnancy-induced cardiac hypertrophy and left ventricular dilation are quickly reversed after birth in absence of lactation in wt mice.

Next, we wanted to assess the cardiac response in *Metrnl*-deficient mice to the non-BF conditions. We found that the circulating levels of Metrnl were significantly reduced in non-BF mice compared to BF mice (**Figure 6D**), but the cardiac mRNA *Metrnl* levels were unchanged suggesting a role of systemic Metrnl during this period (**Figure 6E**). The HW/TL ratio was significantly reduced in non-BF mice compared to BF females in wt and *Metrnl^-/-^* mice by day 14 (**Figure 6F**). Despite this similar trend in decreasing heart weight between genotypes, histological sections from these hearts revealed a larger CSA in *Metrnl^-/-^* mice, even in the absence of lactation (**Figure 6G**). Likewise, we assessed the expression levels of hypertrophy gene markers and, whereas *Acta1* expression was unchanged, *Nppa* expression levels were significantly reduced in heart from no BF wt mice. Conversely, in *Metrnl^-/-^* mice the expression of the hypertrophy markers *Acta1* and *Nppa* remained higher in non-lactating mice (**Figure 6H**). Collectively these data point out to a delay in the reversion of pregnancy-induced cardiac hypertrophy elicited by the absence of lactation in *Metrnl^-/-^* mice.

## Discussion

Here we describe the sequential maternal cardiac events associated with pregnancy, lactation and weaning in mice. We show how the maternal heart develops an adaptive pregnancy-induced cardiac hypertrophy, in agreement with previous reports (4, 8, 9). Such hypertrophy, initiated in pregnancy, further increases in lactation, peaks by day 14 of lactation and starts reverting upon weaning. So far, only a few reports have described post-partum reversibility of gestational cardiac hypertrophy, but the impact of the lactation period has been poorly analyzed (5, 11, 12). In this regard, cardiac fitness after delivery (with or without breastfeeding) has been poorly explored, studies are limited to the characterization in blood flow increase and cardiac output (36), condition probably expected for milk production. One study has found lactation but not parturition key in the onset of postpartum cardiomyopathy in a murine model prone to cardiac dysfunction (37), yet this study does not further explore the mechanism behind the lactation effects in the heart. Our data clearly shows that, during lactation, the hypertrophic condition is exacerbated leading to a more eccentric phenotype by the end of lactation with alterations in the hypertrophic markers. However, this remodeling is completely resolved after weaning. The fact that the key parameters of cardiac function are not altered all over the lactation period and the reversibility of the process agrees with the idea that an intense lactation-associated cardiac hypertrophic phenotype is not pathological, similarly to the hypertrophic remodeling associated to gestation (4, 6).

Our study also shows that METRNL/Metrnl levels increase during pregnancy and decrease during the post-partum period. In mice and humans, this is associated with a huge expression of METRNL/Metrnl in the placental tissue arguing in favor of a placental origin for the high-level of METRNL/Metrnl in plasma during pregnancy. Our data on heterozygous placenta in *Metrnl^-/-^* females strongly supports a role of placental origin of Metrnl during pregnancy. Moreover, in pregnant mice we have also observed increased levels of Metrnl in the myocardium that might also contribute to the circulating levels. We also show how METRNL circulating levels are reduced in obese pregnancies together with a reduced expression of METRNL in the placenta. Human studies have shown that obesity is in general associated with reduced circulating levels of METRNL (38) and to our knowledge only one study has addressed so far, the role of METRNL in human pregnancy showing that maternal obesity decreases Metrnl concentrations in cord blood (39). Furthermore, it has shown that obese women at term have altered cardiac geometry and suboptimal diastolic function (40) and similar findings have been reported in mice subjected to a high-fat diet (41, 42). Considering the cardioprotective role of Metrnl previously described (16, 19) and the adverse cardiac remodeling observed in our *Metrnl*-null mice during pregnancy, our data suggests that increased METRNL/Metrnl levels contribute to the normal performance of the maternal myocardium and the reduced circulating levels of METRNL observed in obese pregnancy may negatively impact the maternal heart.

The positive relationship between high levels of METRNL and mortality associated with cardiovascular disease previously reported (16, 20) may be viewed as a first glance as paradoxical, given that we also found high METRNL/Metrnl levels associated to pregnancy.

However, considering that Metrnl exerts cardioprotection, we can argue that the increased levels of Metrnl achieve full protection during pregnancy but not during the development of cardiac pathology, when high Metrnl levels mainly produced by the myocardium are likely to be due to an unsuccessful protective response, reactive to cardiac compromised function.

In the current study, we show that after the placenta, the maternal myocardium accounts for the highest levels of Metrnl expression (16, 32), suggesting a pivotal role of Metrnl in the maternal heart specially during late pregnancy. In fact, our data points in that direction since most of the cardiac alterations observed in *Metrnl*-null mice start during the gestation period and are not completely reversed in the absence of lactation. Moreover, in the absence of Metrnl, pregnancy exacerbates the cardiac hypertrophic response leading to cardiac disfunction during the lactation period.

Our data reveals that the profile of Metrnl expression in the myocardium parallels the levels of key genes involved in fatty acid metabolism, increasing during the gestation period and dropping post-birth, in agreement with previous studies (8, 11). The observation that Metrnl deficiency impairs myocardial fatty acid metabolism, leading to lipid accumulation, a feature associated with pathological outcomes, without significant changes in inflammatory or angiogenic profiles (17–19), suggests that the disrupted metabolic switch may underlie the adverse cardiac remodeling observed in mice lacking Metrnl, as reported in other models of pathological hypertrophy (16).

Although hypertrophy associated with pregnancy is defined as a physiological process that is usually resolved without complications, pregnancy itself is a stressful situation for the maternal myocardium since it can intensify existing cardiovascular disease and is responsible for the development of two unique disorders, pre-eclampsia and post-partum cardiomyopathy (43, 44). Besides, it has been recently shown that multiparity leads to permanent changes in cardiac structure and some degree of dysfunction (45), and complications during pregnancy impacting the cardiovascular system are associated with increased risk of developing CVD later in life (46). Regarding lactation, the plasticity of maternal heart in this physiological process is poorly understood. Few cohorts have found convincing results on whether mothers with previous CVD should breastfeed or not, probably because most women are advised not to breastfeed due to concerns in drug safety for the baby. However, focusing on the mother’s health, the recommendation has been to opt for an alternative nutritional resource for the babies since the cardiac overload and hormones crucial for milk production, like prolactin, could be determining cardiac recovery after delivery (47). Our data showing altered maternal remodeling when Metrnl levels are low -in Metrnl-null mice and in obese pregnancy- points to Metrnl as a key player for the normal performance of the heart during the gestation and lactation period.

## Conclusions

This study highlights the importance of the gestation-lactation period for maternal cardiovascular health, a subject of utmost interest since the recent proposal of cardio-obstetrics as a new discipline (48). Pregnancy imposes a stressful condition on the maternal myocardium, peaking with adaptive cardiac hypertrophy by day 14 of lactation in murine models before reverting upon weaning, making focused efforts on this period mandatory.

Metrnl emerges as a main actor in these physiological cardiac adaptations during pregnancy and lactation in mice, with low circulating METRNL levels linked to abnormal cardiac function in the last trimester of gestation. This positions METRNL as a potential early diagnostic biomarker for maternal cardiovascular risk, though its regulation during human lactation and impacts on cardiac plasticity require further study.

Proper characterization and follow-up of women’s cardiac adaptations in pregnancy and lactation could enable early identification of cardiovascular diseases in at-risk populations and improve puerperal cardiology management. While this murine study provides a complete picture of the period, additional human research is essential to validate METRNL’s role and prevent future cardiac complications.

## Data Availability

The original contributions presented in the study are included in the article/**Supplementary Material**. Further inquiries can be directed to the corresponding author/s.
