# Supplementary material for: Meteorin-like controls metabolic adaptations and physiological myocardial remodeling in pregnancy and lactation
Source: Front Endocrinol (Lausanne). 2026 Jun 24;17:1823116. doi: 10.3389/fendo.2026.1823116 (PMC13341491; doi:10.3389/fendo.2026.1823116)
Supplement: Supplementary file 1 [file DataSheet1.docx]

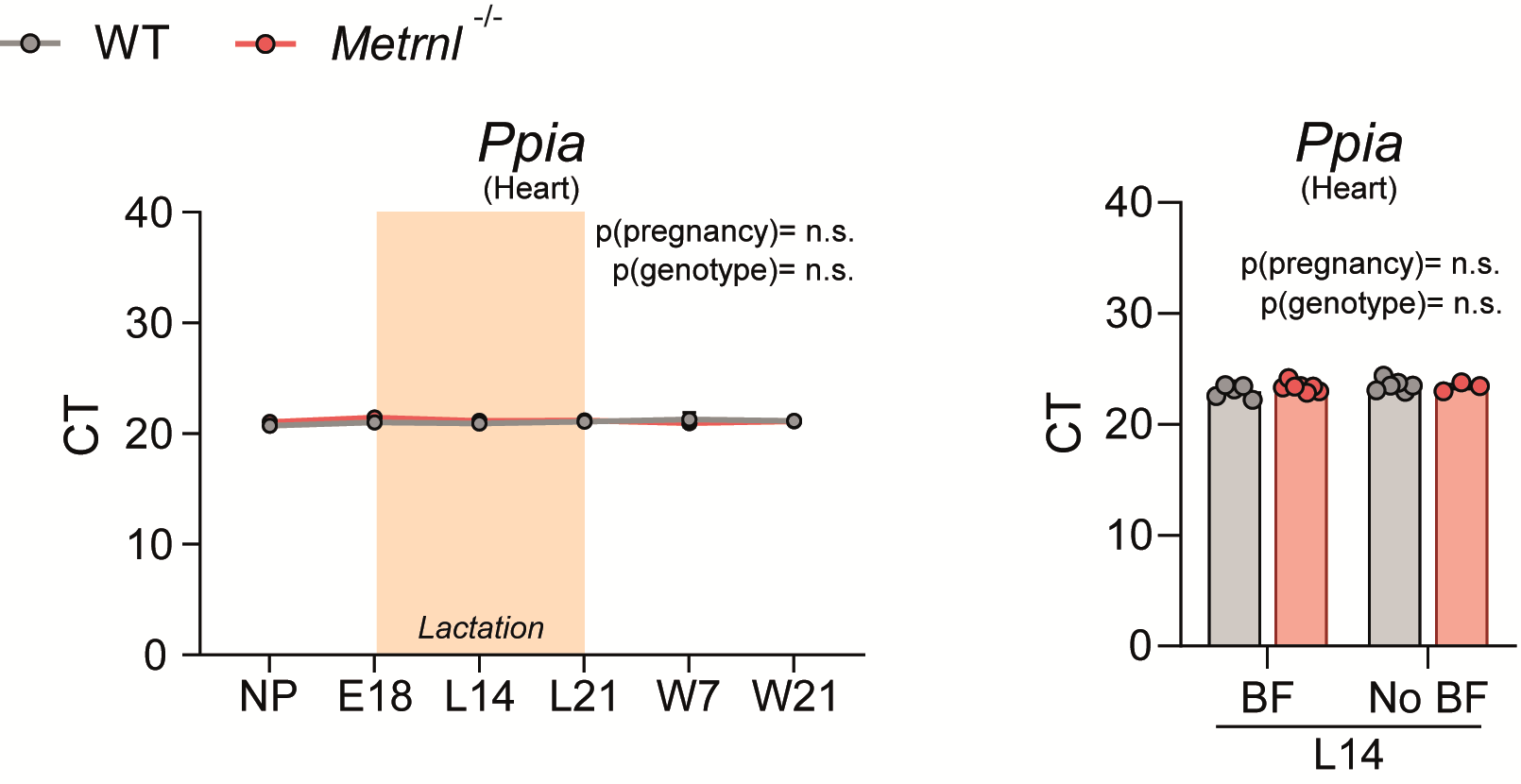


**Supplementary Figure S1. Ct values of the reference gene *Ppia* across genotype and throughout the reproductive cycle.** *Left:* Wild-type and Metrnl-/- female mice were mated overnight. Age-matched non-pregnant (NP) female mice served as controls. NP, pregnant mice at day 18 (E18), lactating mice at days 7 (L7), 14 (L14) and 21 (L21); and mice after weaning at days 7 (W7), 21 (W21) were studied. *Right:* Wild-type (wt) and Metrnl-/- female mice were mated overnight. Upon delivery, a subset of dams had pups removed (non-breastfeeding, non-BF), while the remaining dams breastfed normally (BF).


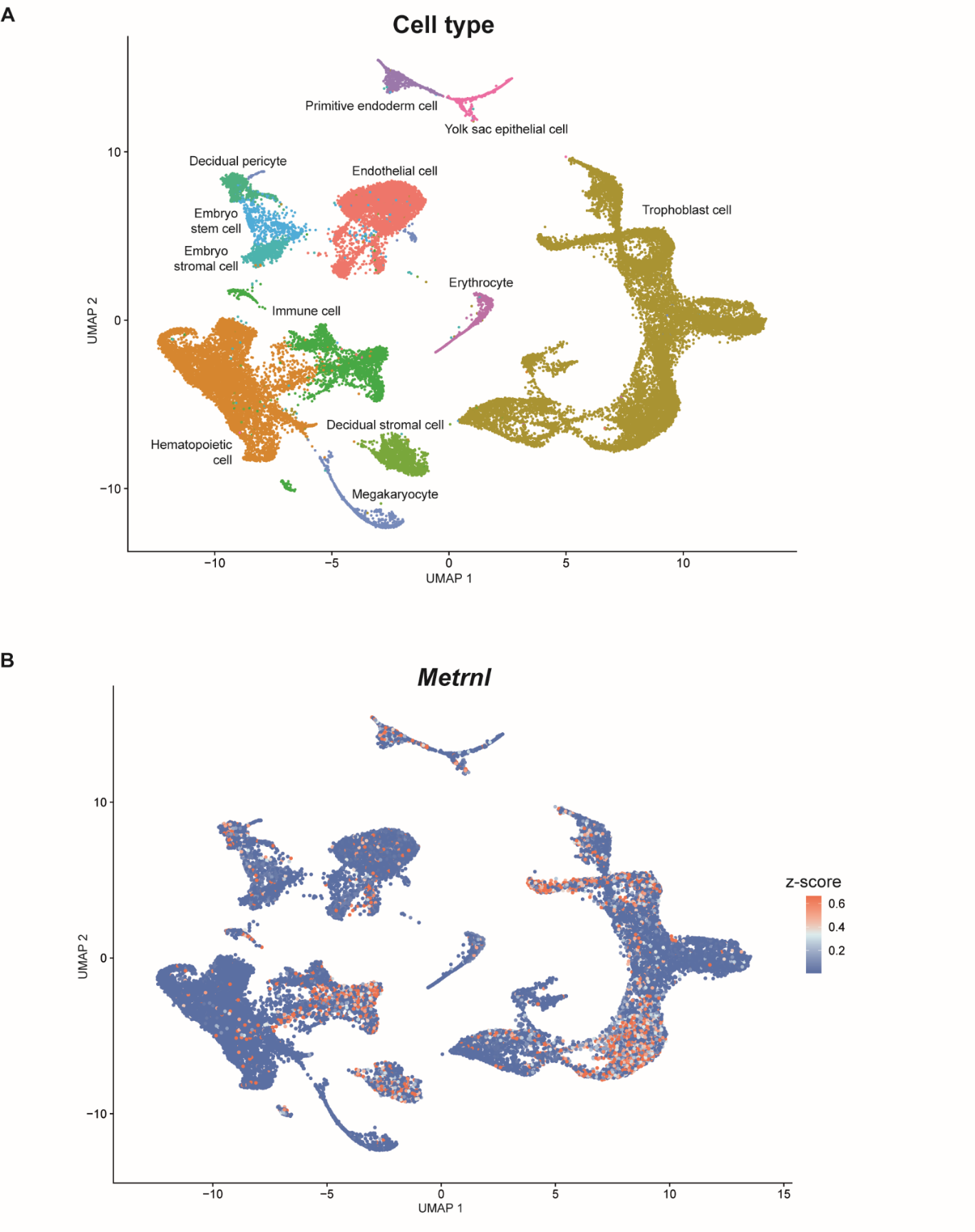


**Supplementary Figure S2. Metrnl expression in murine placenta cell populations.** ScRNA-seq publicly available data (GSE156125) were accessed to characterize Metrnl expression levels. (A) UMAP of placental cells after QC, filtering, integration, and clustering for proper visualization of the cell population clusters is shown and (B) Metrnl expression levels in each cluster are presented.


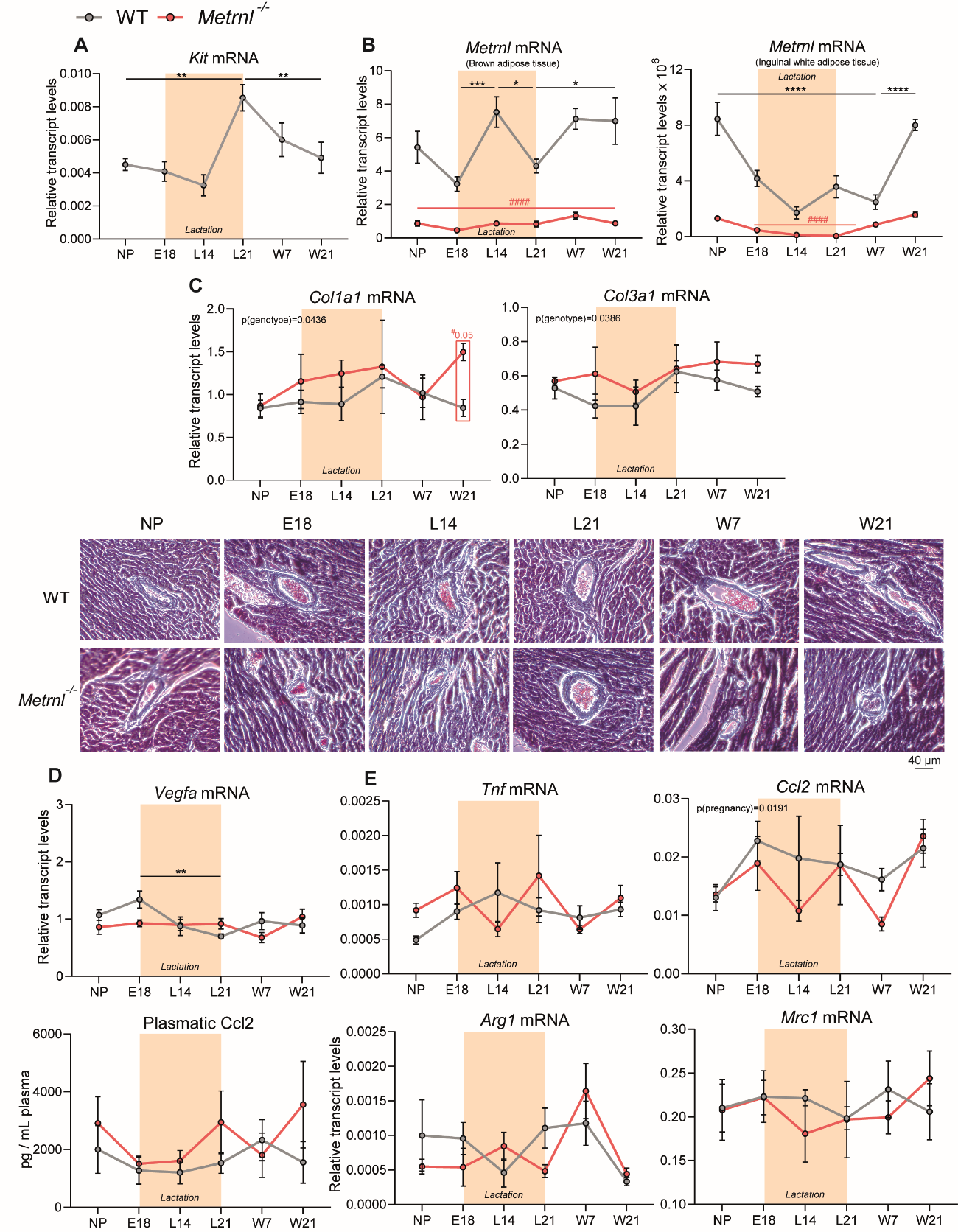


**Supplementary Figure S3. Pregnancy and lactation effects over Kit (Metrnl putative receptor), fibrosis and inflammation gene markers.** Wild-type and Metrnl-/- female mice were mated overnight. Age-matched non-pregnant (NP) female mice served as controls. NP, pregnant mice at day 18 (E18), lactating mice at days 7 (L7), 14 (L14) and 21 (L21); and mice after weaning at days 7 (W7), and 21 (W21) were studied. LV from these hearts was processed for RNA purification. (A) Metrnl receptor, *Kit* (c-Kit) mRNA expression in wild-type mice is shown. (B) *Metrnl* mRNA expression levels in brown and inguinal white adipose tissues in wt and *Metrnl-/-* mice. (C) mRNA expression of fibrosis markers (*Col1a1* and *Col3a1*) wt and *Metrnl-/-* mice and representative histological sections of hearts stained with Masson’s trichrome staining from wild-type (upper panel) and Metrnl-/- (lower panel) from these females are shown (scale bar, 40µm). (D) *Vegfa* mRNA expression. (E) mRNA expression of inflammatory gene markers (*Tnf, Ccl2, Arg1,* and *Mrc1*) and plasmatic levels of Ccl2 in wt and *Metrnl-/-* mice. Results are presented as the mean ± SEM; One-way ANOVA and Sidak’s post-hoc corrections were applied, *p<0.05 and **p<0.01 compared to indicated time-points, same genotype. #p<0.05 for genotype comparisons at a given time-point. Genotype effect is indicated (p(genotype)) at each graph when statistically significant.

**Supplementary Figure S4.** Pregnancy and lactation effects over lipid metabolism marker genes. Wild-type and Metrnl-/- female mice were mated overnight. Age-matched non-pregnant (NP) female mice served as controls. NP, pregnant mice at day 18 (E18), lactating mice at days 7 (L7), 14 (L14) and 21 (L21); and mice after weaning at days 7 (W7), and 21 (W21) were studied. LV from these hearts was processed for RNA purification. mRNA expression levels of canonical lipid metabolism marker genes (*PPARγ, Lpl, Pnpla2*, and *Dgat1*) in wt and Metrnl-/- mice. Results are presented as the mean ± SEM; One-way ANOVA and Sidak’s post-hoc corrections were applied, *p<0.05 and **p<0.01 compared to indicated time-points, same genotype. #p<0.05 for genotype comparisons at a given time-point. Genotype effect is indicated (p(genotype)) at each graph when statistically significant.
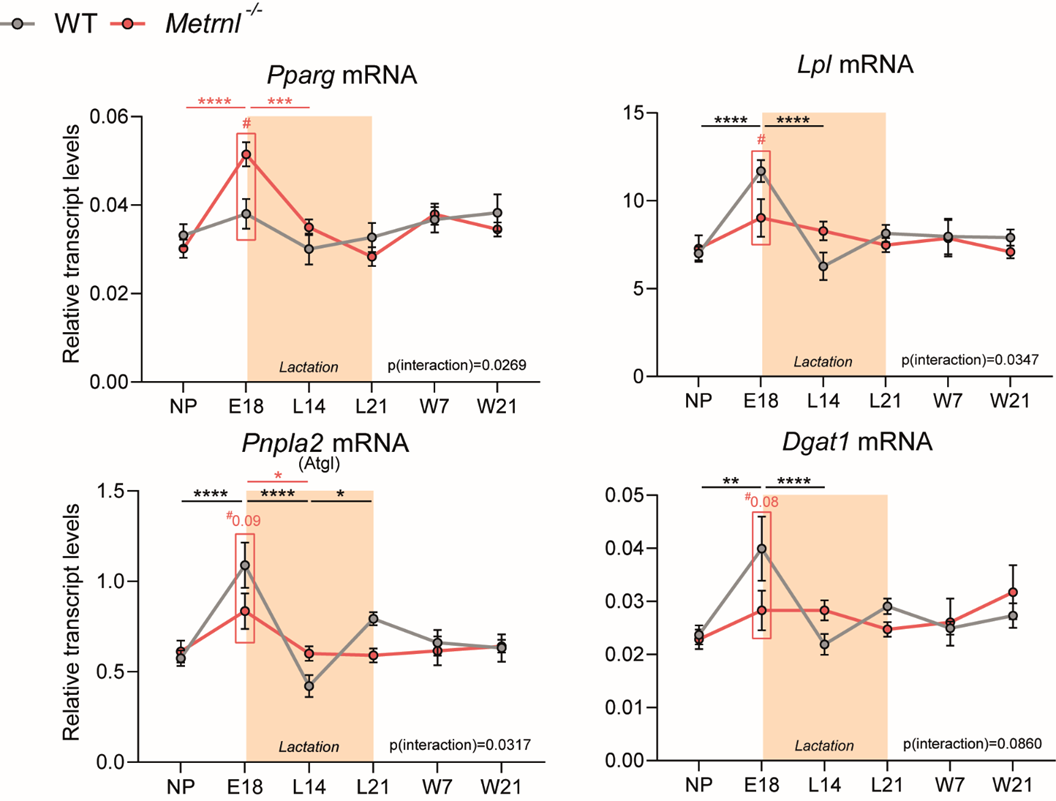


Results are presented as mean + SEM. Data were analyzed by Two-tailed unpaired Student’s t-test.

| Supplementary Table S1. Clinical data of pregnant women at third trimester | | |
| --- | --- | --- |
|  | Non-obese pregnant | Obese pregnant |
| Number | 43 | 58 |
| Age | 30.9±4.7 | 31±3.7 |
| BMI (at term) | 22.5±5.2 | 37.5±2.7^****^ |
| Gestational Age Delivery | 39.4±1.2 | 40±0.66 |
| Gestational Diabetes | 2 | 3 |
| Hypertensive Disease | 0 | 0 |

| **Supplementary Table S2. Genotype data on litter size, pup and placenta morphology**. | | | |
| --- | --- | --- | --- |
|  | **WT** | ***Metrnl^-/-^*** | **p-value** |
| **Pup number** | 6.48 ± 0.43 | 7.00 ± 0.42 | 0.18 |
| **Male pup weight L21** (g) | 9.76 ± 0.57 | 10.14 ± 0.65 | 0.28 |
| **Female pup weight L21** (g) | 8.79 ± 0.38 | 9.08 ± 0.83 | 0.23 |
| **Placenta weight** (mg) | 99.8 ± 4.3 | 93.5 ± 5.42 | 0.36 |

Results are presented as mean + SEM. Data were analyzed by Two-tailed unpaired Student’s t-test. Sample size: litter size n=20-23, pup morphology n=7-14, and placenta morphology n=15-19.

|  | **NP** | | **E18** | | **L7** | | **L14** | |
| --- | --- | --- | --- | --- | --- | --- | --- | --- |
|  | **Wt** | **Metrnl^-/-^** | **Wt** | **Metrnl^-/-^** | **Wt** | **Metrnl^-/-^** | **Wt** | **Metrnl^-/-^** |
| **EDV** (mm^3^) | 56.39 ± 1.39 | 54.77 ± 3.01 | 78.20 ± 3.75** | 66.64 ± 6.00**** | 83.63 ± 2.82*** | 83.44 ± 3.97**** | 93.37 ± 2.44**** | 106.25 ± 5.28****,^####^ |
| **ESV** (mm^3^) | 29.93 ± 1.49 | 26.75 ± 2.12 | 39.10 ± 4.35 | 34.74 ± 3.44 | 42.12 ± 1.63* | 42.19 ± 2.69* | 47.93 ± 2.35* | 55.09 ± 3.91** |
| **LVm** (%) | 100.00 ± 3.82 | 104.08 ± 6.68 | 144.25 ± 7.87** | 162.80 ± 8.80** | 142.51 ± 5.44** | 179.68 ± 13.29** | 179.19 ± 5.11*** | 199.80 ± 7.52***,^##^ |
| **HR** (bpm) | 507.05 ± 24.46 | 473.76 ± 27.93 | 461.86 ± 12.28 | 472.48 ± 11.55 | 484.81 ± 21.81 | 477.10 ± 19.15 | 484.29 ± 17.50 | 490.86 ± 15.95 |
| **Aortic** **peak** (m/s) | 0.72 ± 0.015 | 0.68 ± 0.018 | 0.81 ± 0.039 | 0.81 ± 0.035 | 0.84 ± 0.013* | 0.85 ± 0.025** | 0.89 ± 0.026* | 0.99 ± 0.047** |
| **VTI** (cm) | 3.90 ± 0.104 | 3.56 ± 0.116 | 4.25 ± 0.202 | 4.43 ± 0.346* | 4.20 ± 0.086 | 4.52 ± 0.159 | 4.50 ± 0.138* | 4.87 ± 0.151** |
| **Aortic** **diameter** (mm) | 1.44 ± 0.012 | 1.44 ± 0.007 | 1.64 ± 0.029** | 1.68 ± 0.026** | 1.62 ± 0.018*** | 1.68 ± 0.043* | 1.66 ± 0.020*** | 1.78 ± 0.032*** |

**Supplementary Table S3. Additional echocardiographic parameters for Wt and Metrnl^-/-^ female mice during pregnancy, lactation and weaning.**

|  | **L21** | | **W7** | | **W21** | | **W35** | **W63** |
| --- | --- | --- | --- | --- | --- | --- | --- | --- |
|  | **Wt** | **Metrnl^-/-^** | **Wt** | **Metrnl^-/-^** | **Wt** | **Metrnl^-/-^** | **Metrnl^-/-^** | **Metrnl^-/-^** |
| **EDV** (mm^3^) | 100.01 ± 4.03****,^##^ | 114.59 ± 6.28****,^####^ | 81.64 ± 2.26***,^$^ | 84.92 ± 2.62****,^#^,^$$$$^ | 71.91 ± 5.96,^$$$^ | 84.56 ± 5.31****,^$$$$^ | 86.08 ± 1.57 | 69.43 ± 3.98 |
| **ESV** (mm^3^) | 56.41 ± 2.92** | 69.39 ± 4.09***,^##^ | 46.69 ± 2.54* | 46.93 ± 1.70**,^#^,^$^ | 41.36 ± 4.04 | 46.05 ± 4.30 | 49.44 ± 2.88 | 35.16 ± 4.65 |
| **LVm** (%) | **181.60 ± 5.60***** | **212.47 ± 11.59***,^+^** | 148.68 ± 4.66*** | 152.64 ± 8.11*** | 117.50 ± 6.44*,^$$^ | 143.63 ± 4.11***,^$^ | 155.98 ± 7.68 | 126.95 ± 8.09 |
| **HR** (bpm) | 491.62 ± 17.10 | 449.48 ± 25.60 | 492.52 ± 22.64 | 501.62 ± 16.21 | 518.67 ± 22.68 | 528.57 ± 14.51 | 524.42 ± 41.38 | 592.33 ± 145.87 |
| **Aortic** **peak** (m/s) | 0.82 ± 0.027 | 0.88 ± 0.045 | 0.76 ± 0.030 | 0.75 ± 0.029 | 0.80 ± 0.036 | 0.84 ± 0.049 | 0.79 ± 0.034 | 0.85 ± 0.060 |
| **VTI** (cm) | 4.10 ± 0.163 | 4.49 ± 0.200* | 3.79 ± 0.102 | 3.68 ± 0.16^$$^ | 3.60 ± 0.232^##^ | 3.89 ± 0.18 | 3.65 ± 0.045 | 3.23 ± 0.443 |
| **Aortic** **diameter** (mm) | 1.68 ± 0.019*** | 1.76 ± 0.030*** | 1.57 ± 0.019**,^$$^ | 1.64 ± 0.033* | 1.50 ± 0.032^#^,^$^ | 1.62 ± 0.018***,^$^ | 1.64 ± 0.026 | 1.66 ± 0.042 |

Results are presented as mean ± SEM. Data were analyzed by repeated measures Two-way ANOVA, Tukey’s multiple comparisons test. *p<0.05, **p<0.01, ***p<0.001, ****p<0.0001 vs corresponding NP. ^#^p<0.05, ^##^p<0.01, ^####^p<0.0001 vs corresponding E18. ^$^p<0.05, ^$$^p<0.01, ^$$$^p<0.001, ^$$$$^p<0.0001 vs corresponding L21. +p<0.05 vs Metrnl^-/-^. Sample size: n=7; except for W35 n=4; and W63 n=3.

**Supplementary Table S4. Additional echocardiographic parameters for Wt female, non-breastfeeding mice during and after pregnancy.**

|  | **E18** | **L7** | **L14** |
| --- | --- | --- | --- |
| **EDV** (mm3) | 72.69 ± 4.90 | 70.02 ± 3.16 | 72.38 ± 5.43 |
| **ESV** (mm3) | 35.91 ± 2.71 | 39.06 ± 4.23 | 38.93 ± 4.75 |
| **LVm** (%) | 140.17 ± 7.77 | 115.17 ± 3.01* | 113.60 ± 5.53* |
| **HR** (bpm) | 554.53 ± 27.99 | 509.73 ± 35.61 | 565.47 ± 11.11 |
| **Aortic** **peak** (m/s) | 0.81 ± 0.095 | 0.79 ± 0.049 | 0.70 ± 0.023 |
| **VTI** (cm) | 3.86 ± 0.276 | 3.70 ± 0.130 | 3.39 ± 0.336 |
| **Aortic** **diameter** (mm) | 1.63 ± 0.041 | 1.49 ± 0.021** | 1.42 ± 0.014*** |

Results are presented as mean ± SEM. Data were analyzed by repeated measures One-way ANOVA, Dunnett’s multiple comparisons test. *p<0.05, **p<0.01, ***p<0.001 vs E18. Sample size: n=5.


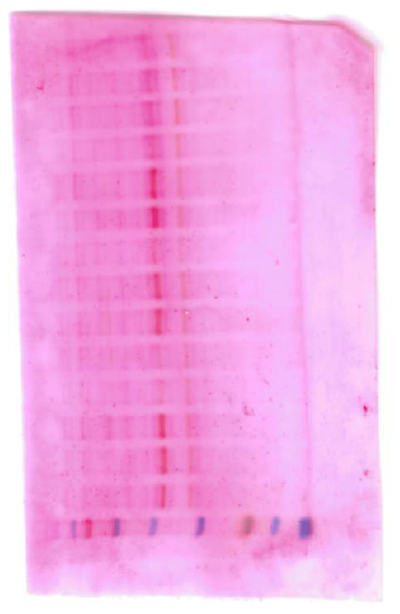
Complete Membranes Figure 4:


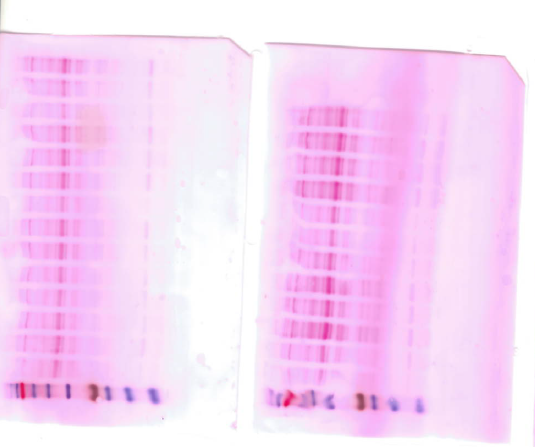

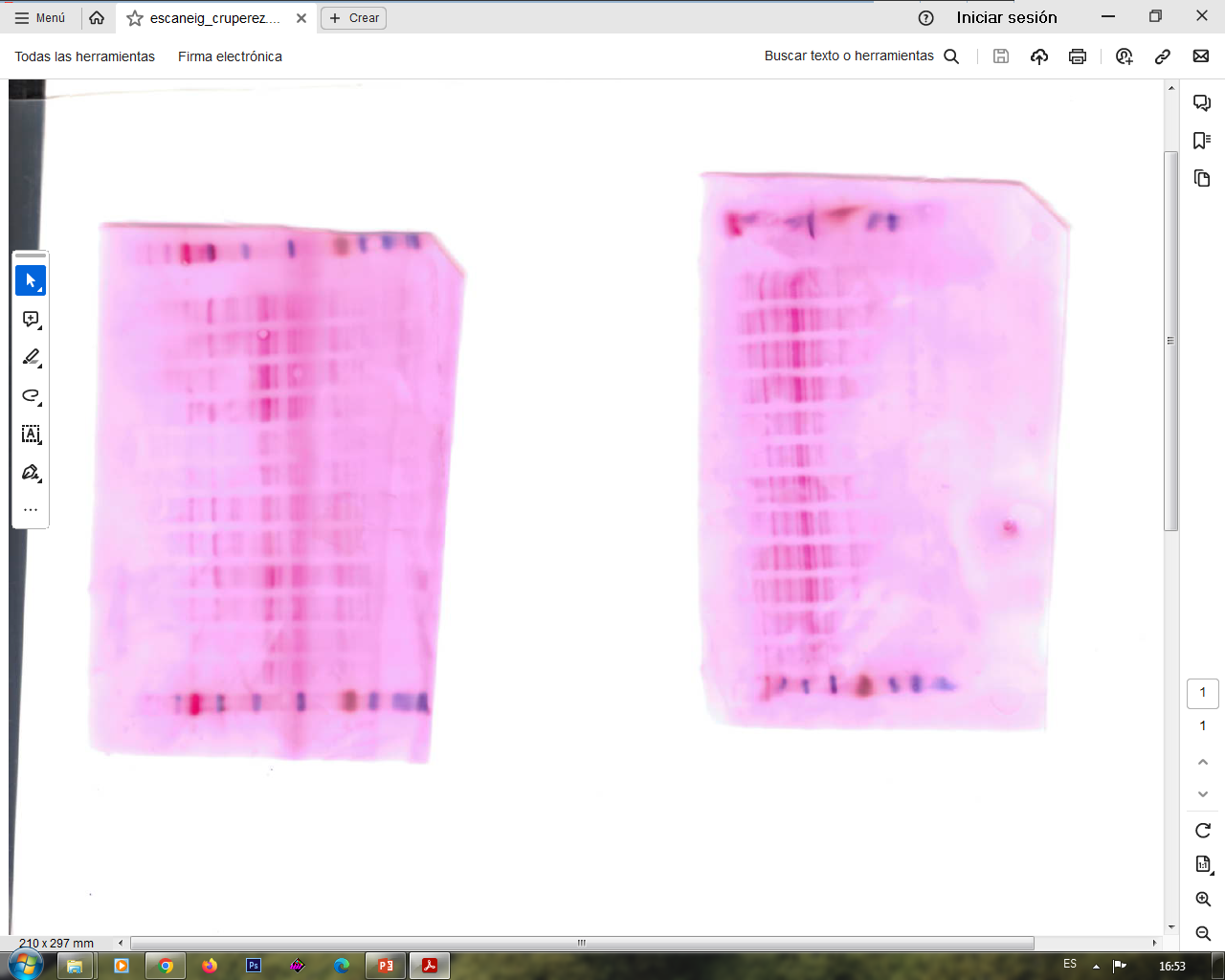

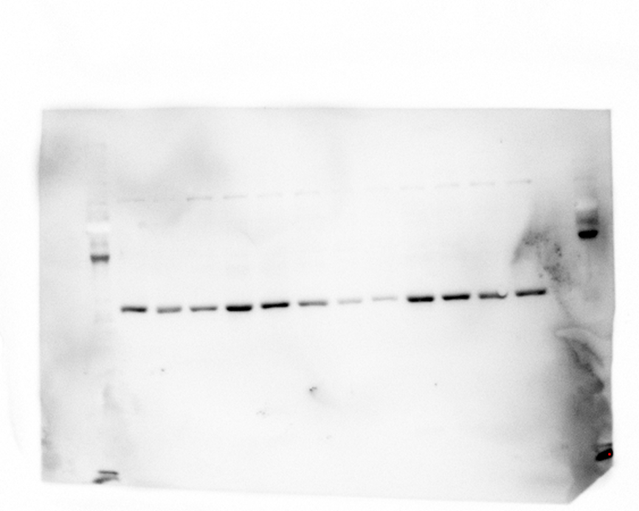

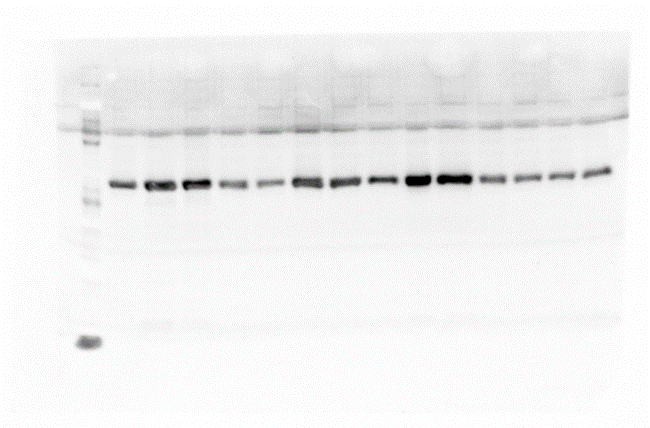
Pdk4 PS

Complete membranes Figure 5:


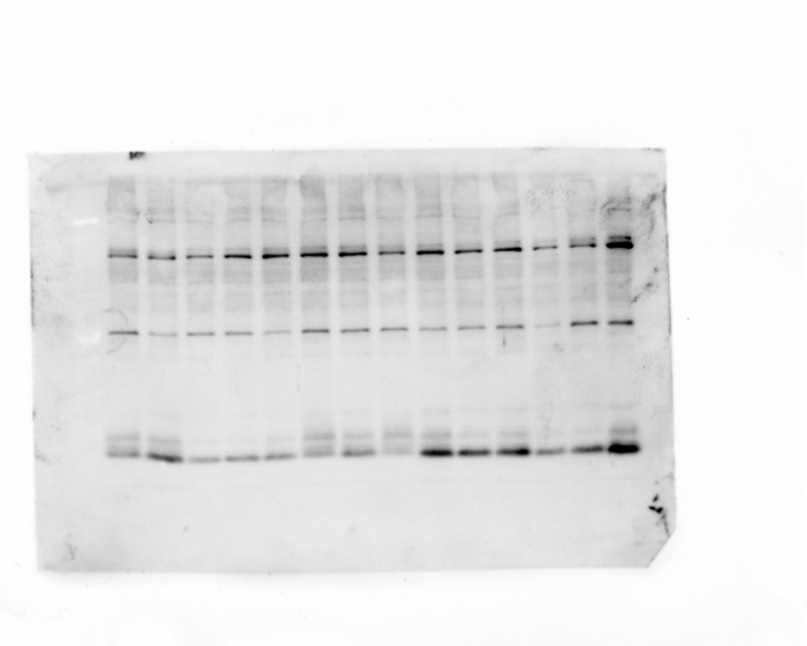
 Mct4 PS


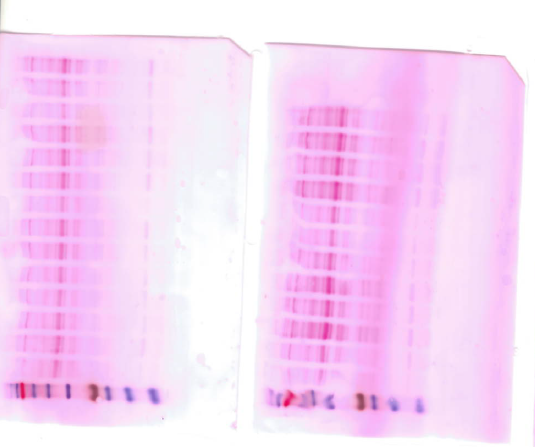


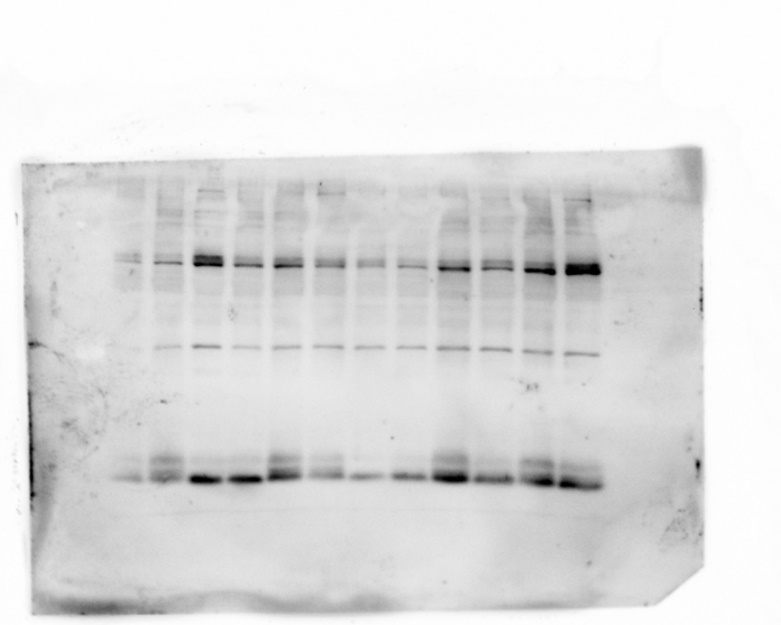


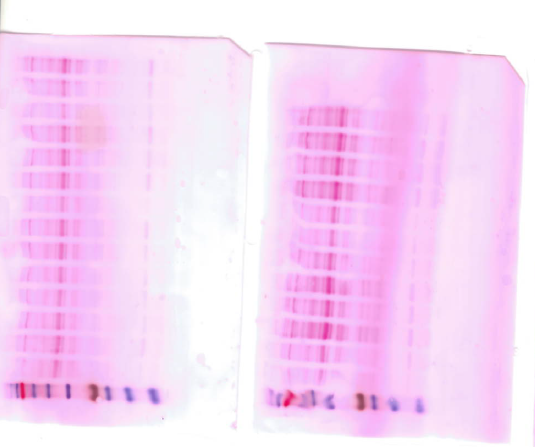

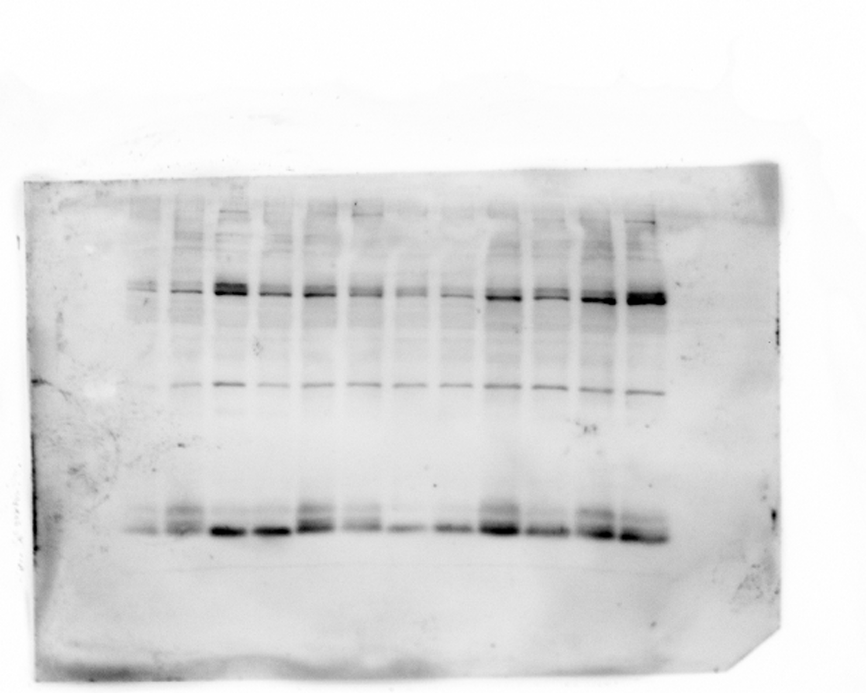


Mct4

L21-W21
